# Causal inference study of plasma proteins and blood metabolites mediating the effect of obesity-related indicators on osteoporosis

**DOI:** 10.3389/fendo.2025.1435295

**Published:** 2025-02-18

**Authors:** Maomao Huang, Fei Xing, Yue Hu, Fuhua Sun, Chi Zhang, Zhangyu Xv, Yue Yang, Qi Deng, Ronglan Shi, Lei Li, Jiayi Zhu, Fangyuan Xu, Dan Li, Jianxiong Wang

**Affiliations:** ^1^ Rehabilitation Medicine Department, The Affiliated Hospital Of Southwest Medical University, Luzhou, China; ^2^ Department of Rehabilitation Medicine, Southwest Medical University, Luzhou, China; ^3^ Rehabilitation Medicine and Engineering Key Laboratory of Luzhou, Luzhou Science and Technology Bureau, Luzhou, China

**Keywords:** obesity-related indicators, osteoporosis, plasma proteins, blood metabolites, mendelian analysis

## Abstract

**Background:**

Osteoporosis and obesity are both major global public health problems. Observational studies have found that osteoporosis might be related to obesity. Mendelian randomization (MR) analysis could overcome the limitations of observational studies in assessing causal relationships.

**Objective:**

This study aims to evaluate the causal potential relationship between obesity-related indicators and osteoporosis by using a two-sample MR analysis and to identify potential mediators.

**Method:**

A total of 53 obesity-related indicators, 3,282 plasma protein lists, and 452 blood metabolite lists were downloaded from the public data set as instrumental variables, and the osteoporosis GWAS data of the MRC IEU Open GWAS database was used as the outcome indicators. Using two-sample univariate MR, multivariate MR, and intermediate MR, the causal relationship and mediating factors between obesity-related indicators and osteoporosis were identified.

**Results:**

The IVW model results show that 31 obesity-related indicators may have a significant causal relationship with osteoporosis (*P* < 0.05), except for waist circumference (id: Ieu-a-71, OR = 1.00566); the remaining 30 indicators could reduce the risk of osteoporosis (OR: 0.983–0.996). A total of 25 plasma protein indicators may have a significant causal relationship with osteoporosis (*P* < 0.05), and 10 of them, such as ANKED46, KLRF1, and LPO, CA9 may have a protective effect on osteoporosis (OR: 0.996–0.999), while the other 15 such as ATP1B1, zinc finger protein 175, could increase the risk of osteoporosis (OR: 1.001–1.004). For blood metabolite indicators, except for alanine (id: Met a-469, OR: 1.071), the other six blood metabolite indicators including uridine and 1-linoleoylglycerophosphoethanolaminecan may have a protective effect on osteoporosis (*P* < 0.05, OR: 0.961–0.992). The direction of causal relationship of MR is all correct; the heterogeneity is all not significant and not affected by horizontal pleiotropy. Using multivariate and mediated MR analysis, it was found that the protective effect of obesity-related indicators against osteoporosis may be mediated by histone-lysine N-methyltransferase in plasma proteins and alanine in blood metabolites.

**Conclusion:**

Obesity may confer a protective effect against osteoporosis, potentially mediated by EHMT2 in plasma proteins and alanine in blood metabolites. Further empirical research is required to fully elucidate the mechanisms behind the influence of obesity on osteoporosis. Interventions on obesity-related factors to reduce the risk of osteoporosis while controlling other adverse effects associated with obesity may require further research.

## 1 Introduction

Osteoporosis is a systemic skeletal degenerative disease characterized by a reduction in bone mass and strength, disruption in the bone microstructure tissue, and increased bone brittleness, which may ultimately lead to osteoporotic fractures (1). The latest studies suggested that more than 200 million people are suffering from osteoporosis worldwide. The prevalence of osteoporosis in people over 50 is about 30% in women and 15% in men (2, 3). This heavy burden of disease could create a huge financial burden. A global study on osteoporosis shows that the average cost of treatment of osteoporosis was US $5,258,741, which accounts for 20% of the GDP of countries in 2018, placing a huge financial burden on health systems (4). Obesity is defined as the excessive or abnormal accumulation and distribution of body fat that poses a threat to health. Over the past few decades, obesity has emerged as an escalating public health concern worldwide (5). The 2017 Global Nutrition Report disclosed that 2 billion adults globally are overweight or obese (6).

Numerous studies have demonstrated a correlation between obesity and osteoporosis. Some research suggests that obesity may have a protective effect against osteoporosis, while other evidence indicates that obesity and the amount of body fat can be risk factors for decreased bone density and fractures (7). A cross-sectional study has revealed a negative correlation between body fat and bone density, indicating that severe obesity is associated with an elevated risk of osteoporosis (8). Zhao et al. demonstrated a negative relationship between fat mass and bone mass after controlling for body mass (9). Kim KC et al. observed that higher body weight and BMI were associated with greater bone mineral density (BMD), potentially lowering the risk of vertebral fractures. Conversely, larger waist circumference and higher body fat percentages were linked to reduced BMD, which could elevate the risk of vertebral fractures (10). However, observational studies have yielded inconsistent findings, and the precise correlation and underlying causality between obesity and its related traits, such as body fat distribution and BMD, require further investigation to be conclusively established.

Mendelian randomization (MR) analysis, which harnesses genetic variation as an instrumental variable, is a robust approach to establish causality between exposures and outcomes in a clinical research (11). Compared with traditional observational studies, MR is less likely to be confounded by external factors (12). A two-sample MR analysis has demonstrated a positive correlation between body mass index (BMI) and BMD at the lumbar spine and heel, yet no such correlation was observed at the femoral neck and forearm (13). That study was limited by its reliance on BMI as the sole indicator of obesity and by its use of data from a single genome-wide association study (GWAS) database. Another recent MR study revealed that different central obesity indicators have different effects on BMD; hip circumference adjusted for BMI showed a negative correlation with BMD, while the waist-to-hip ratio exhibited a positive correlation (14). However, this study also relied on a single-source GWAS database, and the potential mediating effects between obesity and BMD were not further explored.

In addition, osteoporosis is closely related to many factors and biological processes, such as inflammation, estrogen deficiency, cellular senescence, and oxidative stress (15). Certain proteins, including zinc finger protein 267 (ZNF267), ras homologue family member J, actin-binding LIM protein family member 2, programmed cell death 1, and cell cycle protein-dependent kinase-like 5, have been implicated in the pathogenesis of osteoporosis (16, 17). Obesity, as a modulator of bone health, may interact with bone through the endocrine system, adipokines, and inflammation, representing potential mechanisms for bone–adipose tissue crosstalk (18). However, no multifactorial mediated-effect MR studies have yet explored the direct causal link between obesity and osteoporosis nor have they elucidated the underlying mechanisms. Consequently, we initiated a multifactorial MR study to probe the genetic correlations and potential mediators, such as plasma proteins and blood metabolites, between obesity and its associated traits in relation to osteoporosis. This approach aims to provide clearer insights into the complex interplay between obesity and osteoporosis, complementing existing research in the field.

## 2 Method

### 2.1 Study design

**Figure 1:**
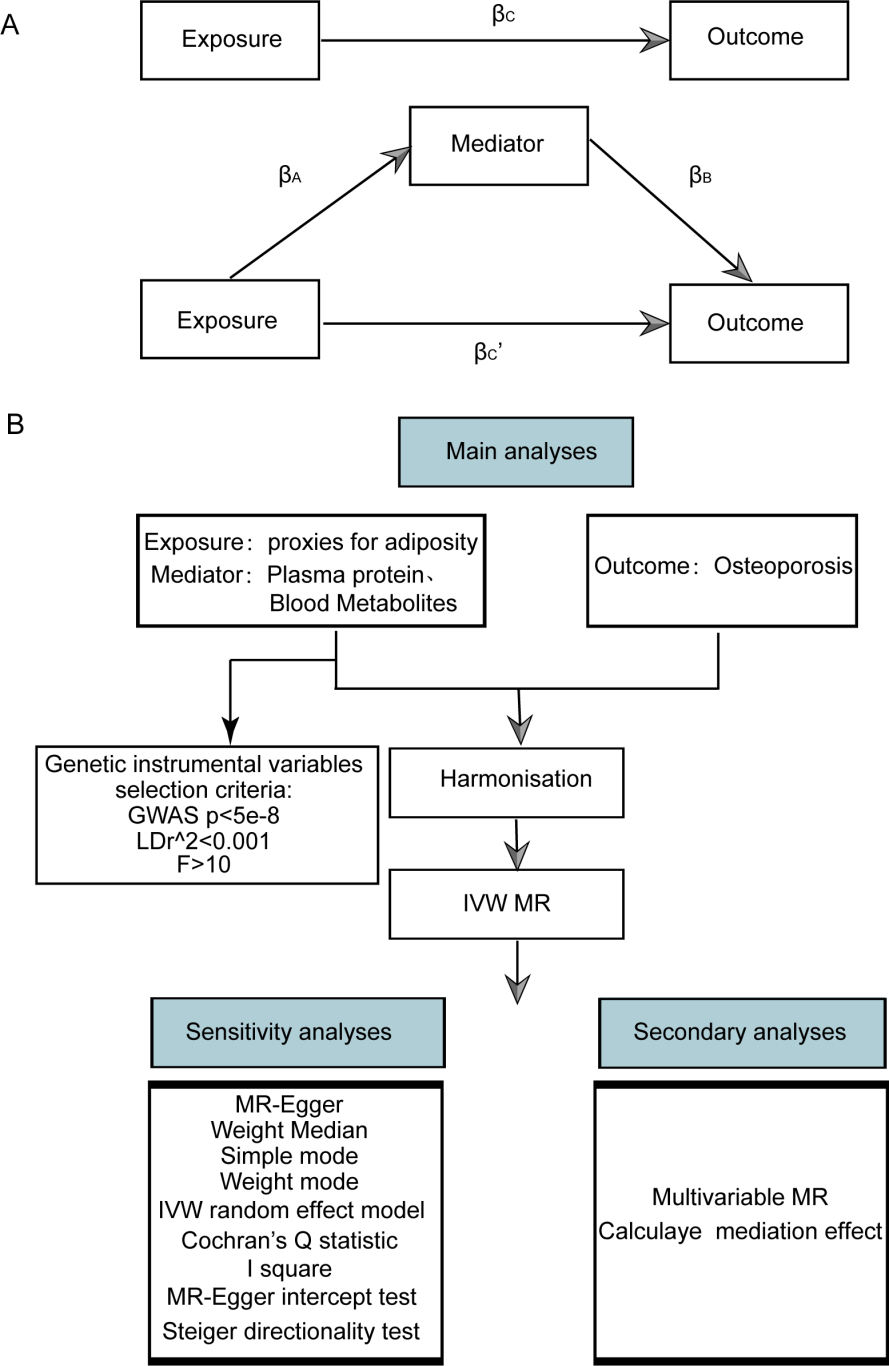
Flow chart of Mendelian randomization analysis. **(A)** Multivariate MR and intermediary role analysis diagram. Basic assumptions of MR. β_A_ is the univariate MR effect value of exposure on mediation, and β_B_ is the direct effect value of exposure on the multivariate MR analysis of outcomes. βc is the univariate MR effect value of exposure on outcomes. βc’ is the multivariable MR Effect value of exposure on outcome. **(B)** Flow chart of analysis methods for this study. SNPs, single-nucleotide polymorphisms; IVW, inverse variance weighted; MR-Egger, Mendelian randomization-Egger; GWAS, genome-wide association study.

We utilized publicly available datasets and two-sample MR to investigate the relationship between adiposity indicators, plasma proteome, and blood metabolites with osteoporosis. Our research methodology and the reporting of results were conducted in strict accordance with the Strengthening the Reporting of Observational Studies in Epidemiology Using Mendelian Randomization (The STROBE-MR Statement) (19). A schematic diagram of our study is shown in **Figure 1**.

### 2.2 Data source

The terms “body mass index”, “waist circumference”, “waist-stature ratio”, “percentage body fat”, “waist-to-hip ratio”, and “fat percentage” were used as keywords to search for obesity-related indicators in the European group from the MRC IEU Open GWAS database (20–22), and 53 obesity-related indicators were obtained (**Supplementary Table S1**). Then TwoSampleMR, a R package, was used to get and standardize the association summary statistics of the 53 indicators.

The list of plasma proteins was obtained from a study on the plasma proteome related to human diseases (23). We queried the MRC IEU Open database using the PMID number 29875488, yielding a dataset of 3,282 plasma proteins (refer to **Supplementary Table S2**). This dataset encompassed data from 3,301 European individuals. The association summary statistics for these proteins were standardized using the TwoSampleMR R package.

The list of metabolites was from a related literature exploring genetic loci affecting human metabolism (24). We conducted a search in the MRC IEU Open database using the PMID number 24816252, obtaining in a dataset comprising 452 blood metabolites (refer to **Supplementary Table S3**). The dataset was derived from 8,242 Europeans. The association summary statistics for these metabolites were standardized by utilizing the TwoSampleMR R package.

The GWAS ID of osteoporosis (ukb-a-87) was obtained from the MRC IEU Open GWAS database. The study cohort was comprised of individuals of European descent, totaling 337,159 samples, which included 5,266 osteoporosis cases and 331,893 controls. The summary statistics for the outcome variable were standardized by utilizing the TwoSampleMR R package.

### 2.3 Instrumental variable selection

A valid genetic instrumental variable must fulfill three core assumptions: (1) the association hypothesis, which states that the chosen instrumental variable must have a notable correlation with the exposure factor; (2) the independence assumption, which dictates that the instrumental variable must not have a significant relationship with any potential confounders that might influence the exposure or the outcome; and (3) the exclusion restriction, which demands that the instrumental variable can only impact the outcome via the pathway of “instrumental variable → exposure → outcome”.

In this study, the criteria of instrumental variable screening for exposure were set as follows: First, a primary screening criterion was set at a *P*-value less than 5 × 10^-8^ for single-nucleotide polymorphisms (SNPs) identified in GWAS. Second, SNPs in linkage disequilibrium, with *r^2^* value of less than 0.001 and separated by a physical distance exceeding 10,000 kb, were excluded. Based on the filtered SNPs, instrumental variables were extracted from the GWAS outcome data. To evaluate potential bias due to weak instrumental variables, F-statistics were computed. When *F* < 10, it suggests that the genetic variation is a weak instrumental variable and might cause a certain bias in the results (25). Therefore, it is necessary to eliminate such variables to prevent affecting the outcomes. The F-statistic calculation formula is as follows:


$$\text{F}=\frac{N-k-1}{k}\times \frac{R^{2}}{1-R^{2}}$$

In this context, *N* represents the sample size, *k* denotes the quantity of instrumental variables employed, and *R* ^2^ reflects the degree to which these instrumental variables account for the exposure. Specifically, *R* ^2^ is calculated using the formula *R* ^2^ = 2 × (1 - MAF) × MAF × 2*β*, where MAF stands for minimum allele frequency and β represents the allele effect size.

### 2.4 MR causal effect estimation

We used a variety of methods of two-sample MR analysis to evaluate the causal effect of obesity-related indicators, plasma proteins, and blood metabolites on osteoporosis, including inverse variance weighted (IVW) (26), weight median (27), MR-Egger (28), and weighted model (29). Under certain conditions, the IVW method is considered marginally more robust than other methods; its distinctive feature is the exclusion of the intercept term during regression and the use of the inverse of the outcome variance as the fitting weight. Therefore, in scenarios where pleiotropy is absent, regardless of heterogeneity, the IVW method serves as the primary MR analysis (utilizing the IVW random-effects model in cases of heterogeneity), complemented by four additional methods. In the presence of pleiotropy, the MR-Egger method is adopted to calculate the results. Finally, we determined the direction of causality through the Steiger test of the TwoSampleMR.

### 2.5 Sensitivity analysis

The sensitivity analysis of the findings was conducted using three tests:

(1) Heterogeneity test: The Cochran Q test was employed to assess the heterogeneity among SNP estimations. A statistically significant Cochran Q test indicates significant heterogeneity in the analysis outcomes. However, this test only determines the presence or absence of heterogeneity; it does not assess its distribution. Therefore, the *I* ^2^ statistic was introduced to represent the proportion of heterogeneity within the instrumental variables relative to the total variation. Specifically, the *I* ^2^ value of ≤0 is set to 0, signifying no heterogeneity; values between 0% and 25% indicate mild heterogeneity, 25%–50% suggest moderate heterogeneity, and >50% denote high heterogeneity. The formula for this calculation is detailed below:


$$I^{2}=\frac{Q-dl}{Q}\times 100\%$$

(2) Pleiotropy test: To assess pleiotropy, the MR-Egger method was applied. A statistically significant MR-Egger intercept with a *P*-value below 0.05 indicates substantial horizontal pleiotropy of the genetic variation.

(3) Leave-one-out test: The leave-one-out test was conducted by iteratively excluding individual SNPs to compute the MR results using the remaining instrumental variables. This was done to evaluate the impact of each SNP on the relationship between exposure factors and outcomes. A significant disparity between the MR effect estimates and the overall effect estimates upon excluding a specific instrumental variable would suggest that the MR effect estimates are highly sensitive to that particular SNP.

### 2.6 Multivariate MR analysis and mediating effect estimation

Multivariable Mendelian randomization (MVMR) extends the traditional MR framework. It utilizes genetic variations linked to several potentially interconnected exposures to ascertain the impact of multiple exposures on a single outcome. This approach enables the assessment of the direct effects of an individual exposure on a specific outcome. Before conducting MVMR, we selected those obesity-related indicators, plasma proteins, and blood metabolites with significant causal effects on osteoporosis in univariate MR as subsequent MVMR exposures. The MVMR models for obesity-related indicators, plasma proteins, and blood metabolites on osteoporosis were constructed for MVMR analysis. The direct effects of obesity-related indicators, plasma protein, and blood metabolites on osteoporosis were obtained through MVMR analysis, and the effects of obesity-related indicators on plasma proteins and blood metabolites were obtained through univariate MR. This allowed us to estimate the indirect effects of obesity-related indicators → plasma proteins or blood metabolites → osteoporosis pathway (**Figure 1**). Effect sizes and standard errors for mediating effects were calculated according to the following equation:

$\beta_{\text{M}}=\beta_{\text{A}}\times \beta_{\text{B}}$


$${\text{SE}}_{\text{M}}=\sqrt{(\beta_{\text{A}}\times {\text{SE}}_{\text{B}}{)}^{2}+(\beta_{\text{B}}\times {\text{SE}}_{\text{A}}{)}^{2}}$$

where $\beta_{M}\;$ is the effect size of mediating effect, and SEM is its corresponding standard error. $\beta_{\text{A}}$ is the univariate MR effect of exposure (obesity-related indicators) on mediator, and SEA is its corresponding standard error. $\beta_{\text{B}}$ is the direct effect of plasma proteins or blood metabolites on osteoporosis (obtained by MVMR), and SEB is its corresponding standard error (**Figure 1A**).

Combined with the causal stepwise regression method, if both $\beta_{\text{A}}$ and $\beta_{\text{B}}$ are significant, the indirect effect is significant. If $\beta_{\text{A}}$ or $\beta_{\text{B}}$ are not significant, Sobel test was used to determine whether $\beta_{\text{M}}$ is significant. If $\beta_{\text{M}}$ is significant, the indirect effect is significant. Under the premise of significant indirect effect, if the MR effect value $\beta_{\text{C}}$ , of obesity-related indicators on osteoporosis in MVMR is significant, the direct effect is significant, and other mediators might exist. Otherwise, the direct effect is not significant, and the complete mediating effect is assumed. Under the premise that both indirect and direct effects are significant, if $\beta_{\text{M}}$ and $\beta_{\text{C}}$ , have different signs, according to the cover effect theory, calculate the covering ratio: | $\beta_{\text{M}}$ / $\beta_{\text{C}}$ , | × 100%. If $\beta_{\text{M}}$ and $\beta_{\text{C}}$ , have the same number, according to the partial mediation effect theory, calculate the mediation accounted for: $\beta_{\text{M}}$ / $\beta_{\text{C}}$ × 100%, where $\beta_{\text{C}}$ is the effect size of obesity-related indicators on osteoporosis in univariate MR. Due to the complexity of the mediating effect, this paper only discusses the mediation effect based on the case where there is a significant causal correlation between exposure and outcome, and there is also a significant causal correlation between exposure and mediating factors.

### 2.7 Statistical analysis

All data computations and statistical analyses were conducted using R (https://www.r-project.org/, version 4.3.1). Primarily, the TwoSampleMR package facilitated MR analysis. To assess the robustness and reliability of the findings, we employed the Cochran Q test and leave-one-out analysis. Genetic pleiotropy was tested using the MR-Egger intercept method. Our evaluation criteria included the odds ratio (OR) and 95% confidence interval (95% CI). All reported *P*-values were two-sided. In the context of SNPs derived from GWAS studies, a *P*-value less than 5 × 10^-8^ was considered statistically significant. For other statistical assessments, a *P*-value below 0.05 was considered statistically significant.

## 3 Results

### 3.1 Analysis framework and flow chart

The MVMR and intermediary role analysis diagram is shown in **Figure 1A**. The flow chart of analysis methods for this study is shown in **Figure 1B**.

### 3.2 Instrumental variable screening

SNPs with linkage disequilibrium were removed according to the screening criteria of instrumental variables in our study. After matching with the GWAS data for osteoporosis, SNPS related to obesity-related indicators, plasma proteins, and blood metabolites were included as instrumental variables. The instrumental variables with a significant *p*-value (<0.05) identified by MR analysis of each index are shown in **Supplementary Tables S4**-**S6**. The F-test statistics for these indicators are greater than 10, suggesting that the screened SNPs had a strong effect and that the potential bias due to weak instrumental variables is limited.

### 3.3 MR causal effect estimates

Five models, including MR Egger, weighted median, IVW, simple mode (SM), and weighted mode were used for analysis. The significance of the IVW model (*P* < 0.05) served as the screening condition for significant causality. The causal effect estimation results for these five models are shown in **Supplementary Tables S7**-**S9**.

**Figure 2:**
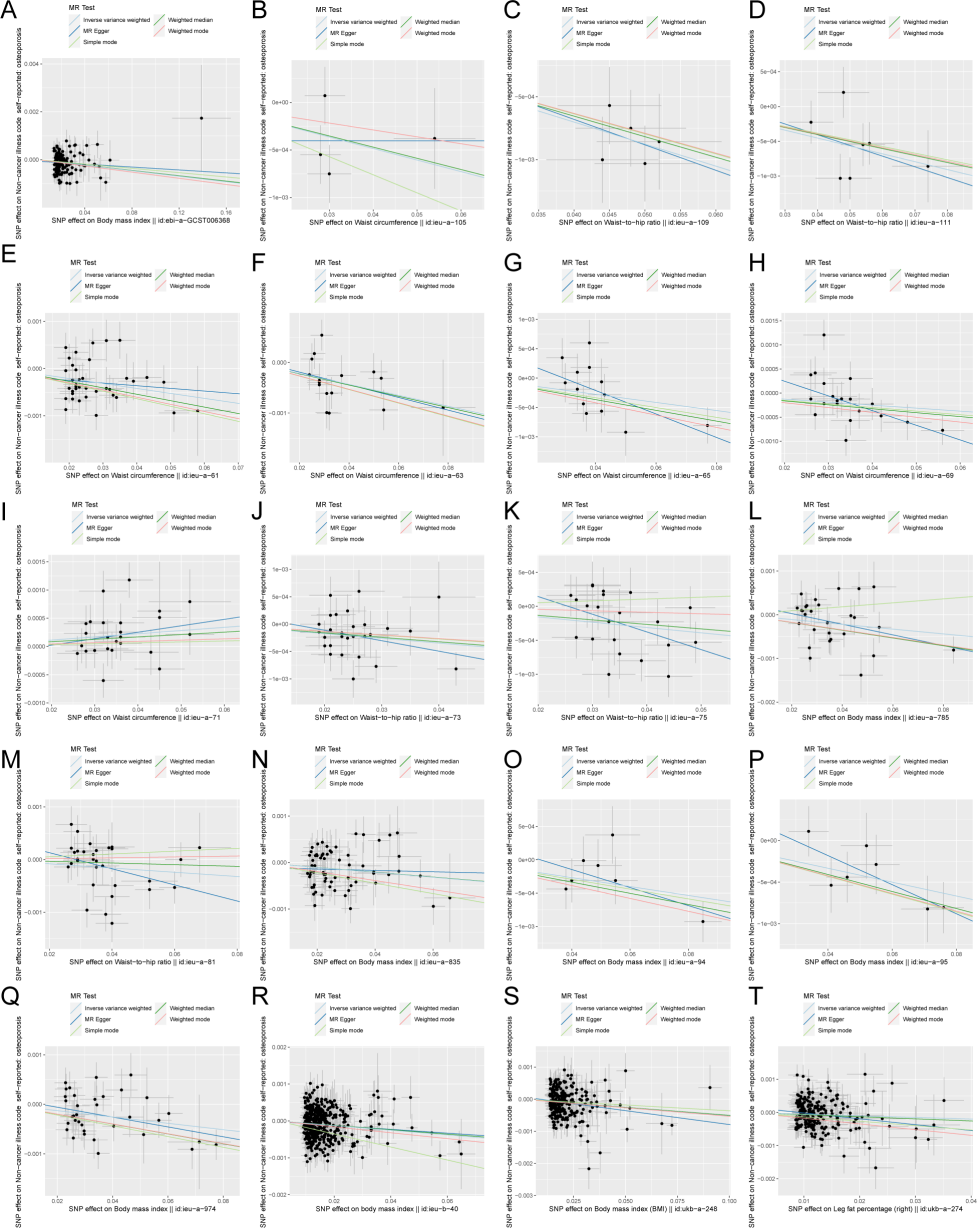
Scatter plot of correlation between obesity-related indicators (part 1) and osteoporosis. **(A)** Body mass index||id: ebi-A-GCST006368. **(B)** Waist circumference||id: ieu-A-105. **(C)** Waist-to-hip the wire||id: Ieu-a-109. **(D)** Waist-to-hip the wire||id: ieu-a-111. **(E)** Waist circumference||id: ieu-a-61. **(F)** Waist circumference||id: Ieu-a-63. **(G)** Waist circumference||id: ieu-a-65. **(H)** Waist circumference||id: ieu-a-69. **(I)** Waist circumference||id: Ieu-a-71. **(J)** Waist-to-hip the wire||id: ieu-a-73. **(K)** Waist-to-hip the wire||id: ieu-a-75. **(L)** Body mass index||id: Ieu-a-785. **(M)** Waist-to-hip the wire||id: ieu-a-81. **(N)** Body mass index||id: ieu-a-835. **(O)** Body mass index||id: Ieu-a-94. **(P)** Body mass index||id: ieu-a-95. **(Q)** Body mass index||id: ieu-a-974. **(R)** Body mass index||id: Ieu-b-40. **(S)** Body mass index (BMI)||id: ukb-a-248. **(T)** Leg fat percentage (right)||id: ukb-a-274. Light blue, IVW; green, weight median; dark blue, MR-Egger; light green, simple mode; pink, weighted model.

**Figure 3:**
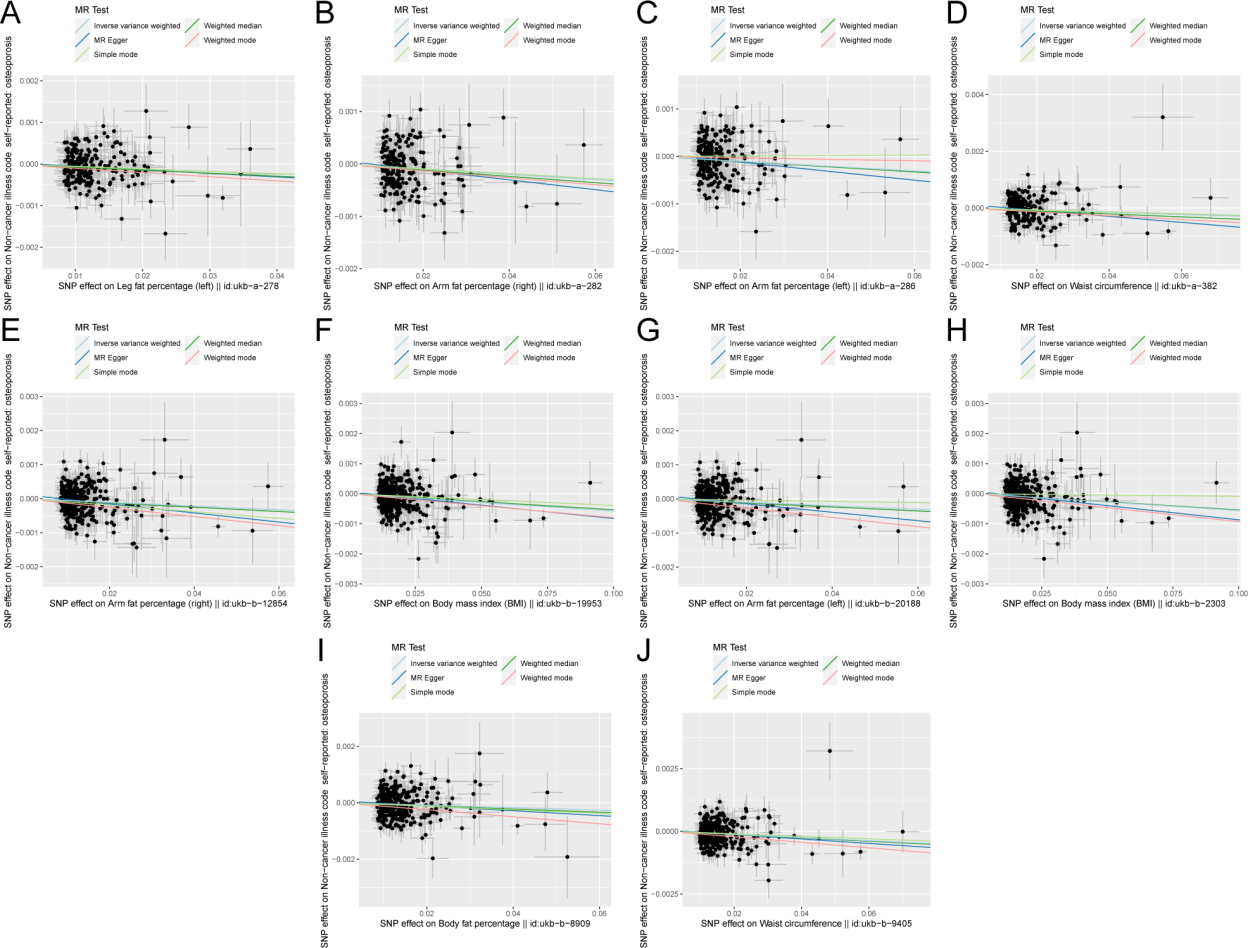
Scatter plot of correlation between obesity-related indicators (part 2) and osteoporosis. **(A)** Leg fat percentage (left)||id: ukb-A-278. **(B)** Arm fat percentage (right)||id: Ukb-a-282. **(C)** Arm fat percentage (left)||id: ukb-a-286. **(D)** Waist circumference||id: Ukb-a-382. **(E)** Arm fat percentage (right)||id: ukb-b-12854. **(F)** Body mass index (BMI)||id: Ukb-b-19953. **(G)** Arm fat percentage (left)||id: ukb-b-20188. **(H)** Body mass index (BMI)||id: Ukb-b-2303. **(I)** Body fat percentage with||id: ukb-b-8909. **(J)** Waist circumference||id: ukb-b-9405). Light blue, inverse variance weighted (IVW); green, weight median; dark blue, MR-Egger; light green, simple mode; pink: weighted model.

**Figure 4:**
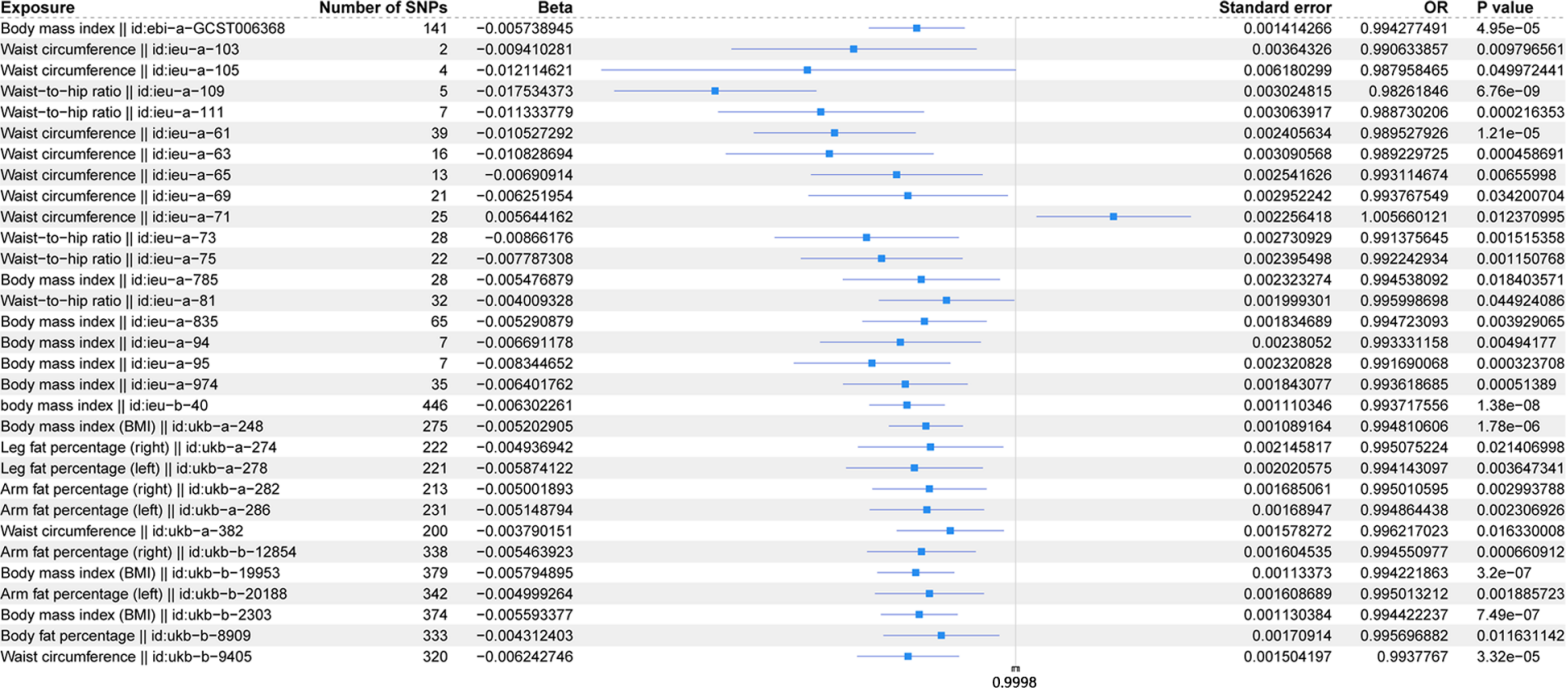
Forest plot of IVW model for MR of obesity-related indicators on osteoporosis.

The scatter plots of the effect estimate for SNPs screened after MR analysis of obesity-related indicators and osteoporosis are shown in **Figures 2**, **3**. Only results with more than two SNPS are shown in the figures. It can be seen that the direction of the scatter plot fitting curve for the five models is essentially the same, and the slopes of most models is relatively consistent. The intercept of the IVW model was close to 0. For obesity-related indicators, the results of the IVW model are shown in **Table 1** and **Figure 4**. The IVW model results show that obesity-related indicators such as BMI, waist circumference, waist-to-hip ratio, leg fat percentage (right), leg fat percentage (left), arm fat percentage (right), arm fat percentage (left), and body fat percentage have a significant causal relationship with the pathogenesis of osteoporosis (*P* < 0.05). Finally, the Steiger test suggested that the causal direction from obesity-related indicators to osteoporosis is correct (**Supplementary Table S10**). The Steiger directionality test calculated the variance rate (*r* ^2^) of SNPs for exposure and outcome, respectively. The results showed that the SNPs for our selected indicators explained more variance of exposure than in outcome, with the direction being TRUE, and the *p*-values were less than 0.05, indicating that the direction is correct.

**Table 1:** Estimation of MR causal effects of obesity-related indicators on osteoporosis (IVW model).

Exposure	ID	Number of SNPs	*β*	Standard error	OR (95%CI)	*p*-value
Body mass index	ebi-a-GCST006368	141	-0.00574	0.001414	0.994277 (0.991525, 0.997037)	0.00005
Waist circumference	ieu-a-103	2	-0.00941	0.003643	0.990634 (0.983585, 0.997733)	0.009797
Waist circumference	ieu-a-105	4	-0.01212	0.00618	0.987958 (0.976063, 0.999999)	0.049972
Waist-to-hip ratio	ieu-a-109	5	-0.01753	0.003025	0.982618 (0.976810, 0.988461)	0
Waist-to-hip ratio	ieu-a-111	7	-0.01133	0.003064	0.988730 (0.982810, 0.994686)	0.000216
Waist circumference	ieu-a-61	39	-0.01053	0.002406	0.989528 (0.984873, 0.994205)	0.000012
Waist circumference	ieu-a-63	16	-0.01083	0.003091	0.989230 (0.983256, 0.995240)	0.000459
Waist circumference	ieu-a-65	13	-0.00691	0.002542	0.993115 (0.988180, 0.998074)	0.00656
Waist circumference	ieu-a-69	21	-0.00625	0.002952	0.993768 (0.988034, 0.999535)	0.034201
Waist circumference	ieu-a-71	25	0.005644	0.002256	1.005660 (1.001222, 1.010118)	0.012371
Waist-to-hip ratio	ieu-a-73	28	-0.00866	0.002731	0.991376 (0.986083, 0.996696)	0.001515
Waist-to-hip ratio	ieu-a-75	22	-0.00779	0.002395	0.992243 (0.987595, 0.996913)	0.001151
Body mass index	ieu-a-785	28	-0.00548	0.002323	0.994538 (0.990020, 0.999077)	0.018404
Waist-to-hip ratio	ieu-a-81	32	-0.00401	0.001999	0.995999 (0.992103, 0.999909)	0.044924
Body mass index	ieu-a-835	65	-0.00529	0.001835	0.994723 (0.991153, 0.998307)	0.003929
Body mass index	ieu-a-94	7	-0.00669	0.002381	0.993331 (0.988707, 0.997977)	0.004942
Body mass index	ieu-a-95	7	-0.00835	0.002321	0.991690 (0.987189, 0.996211)	0.000324
Body mass index	ieu-a-974	35	-0.0064	0.001843	0.993619 (0.990036, 0.997215)	0.000514
body mass index	ieu-b-40	446	-0.0063	0.00111	0.993718 (0.991557, 0.995883)	0
Body mass index	ukb-a-248	275	-0.0052	0.001089	0.994811 (0.992689, 0.996937)	0.000002
Leg fat percentage (right)	ukb-a-274	222	-0.00494	0.002146	0.995075 (0.990899, 0.999269)	0.021407
Leg fat percentage (left)	ukb-a-278	221	-0.00587	0.002021	0.994143 (0.990214, 0.998088)	0.003647
Arm fat percentage (right)	ukb-a-282	213	-0.005	0.001685	0.995011 (0.991730, 0.998302)	0.002994
Arm fat percentage (left)	ukb-a-286	231	-0.00515	0.001689	0.994864 (0.991576, 0.998164)	0.002307
Waist circumference	ukb-a-382	200	-0.00379	0.001578	0.996217 (0.993140, 0.999304)	0.01633
Arm fat percentage (right)	ukb-b-12854	338	-0.00546	0.001605	0.994551 (0.991428, 0.997684)	0.000661
Body mass index	ukb-b-19953	379	-0.0058	0.001134	0.994222 (0.992015, 0.996434)	0
Arm fat percentage (left)	ukb-b-20188	342	-0.005	0.001609	0.995013 (0.991881, 0.998155)	0.001886
Body mass index	ukb-b-2303	374	-0.00559	0.00113	0.994422 (0.992221, 0.996628)	0.000001
Body fat percentage	ukb-b-8909	333	-0.00431	0.001709	0.995697 (0.992367, 0.999038)	0.011631
Waist circumference	ukb-b-9405	320	-0.00624	0.001504	0.993777 (0.990851, 0.996711)	0.000033

SNP, single-nucleotide polymorphism; OR, odds ratio; CI, confidence interval.

**Figure 5:**
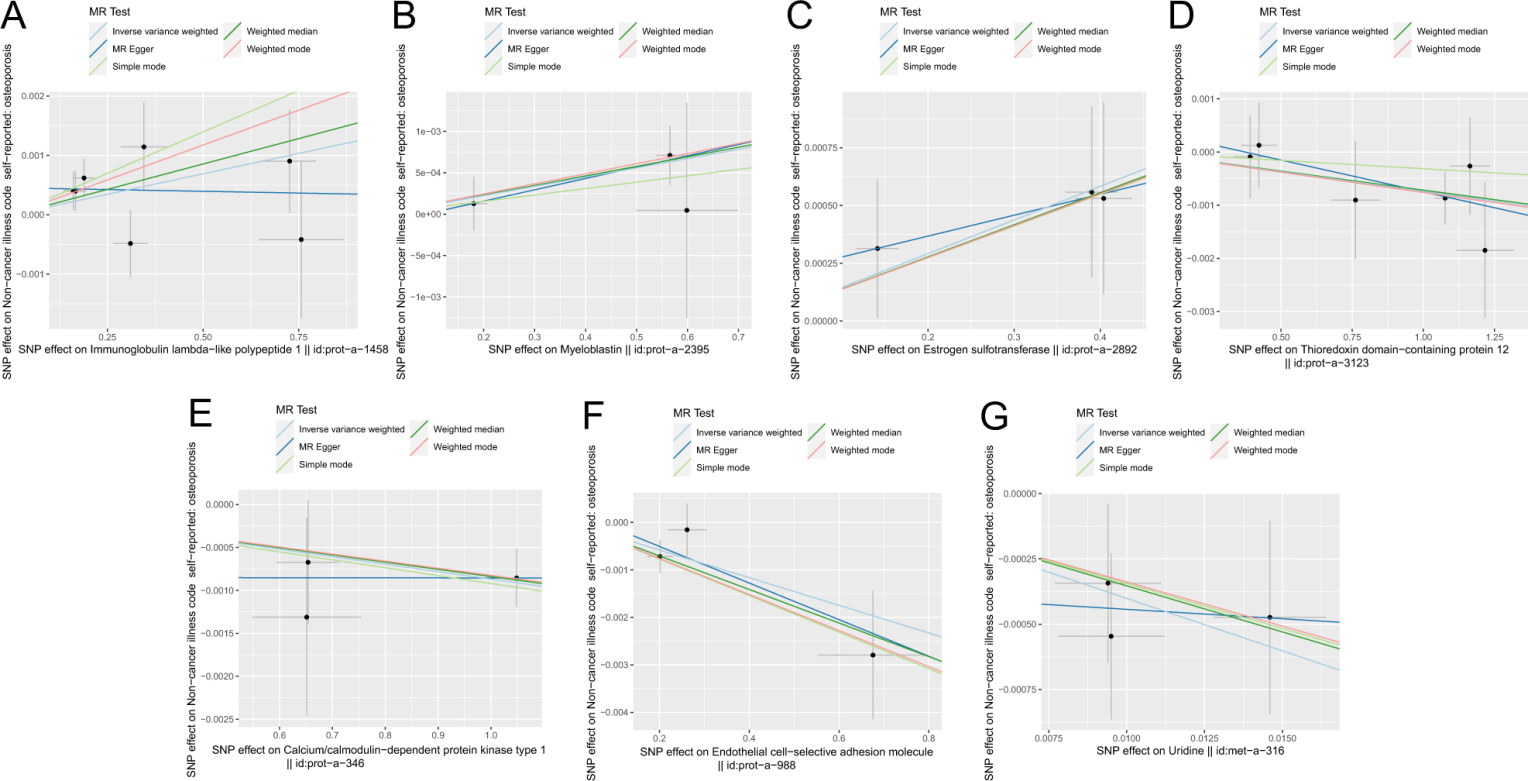
Scatter plot of correlation between plasma proteins, blood metabolites, and osteoporosis. **(A)** Plasma proteins in immunoglobulin lambda-like polypeptide 1||id: prot-A-1458. **(B)** Myeloblastin||id: Prot-a-2395. **(C)** Estrogen sulfotransferase||id: prot-a-2892. **(D)** Thioredoxin domain-containing protein 12||id: Prot-a-3123. **(E)** Calcium/calmodulin-dependent protein kinase type 1||id: Prot-a-346. **(F)** Endothelial cell-selective adhesion molecule||id: prot-a-988. **(G)** Uridine||id: met-a-316.) Light blue, IVW; green, weight median; dark blue, MR-Egger; light green, simple mode; pink, weighted model.

**Figure 6:**
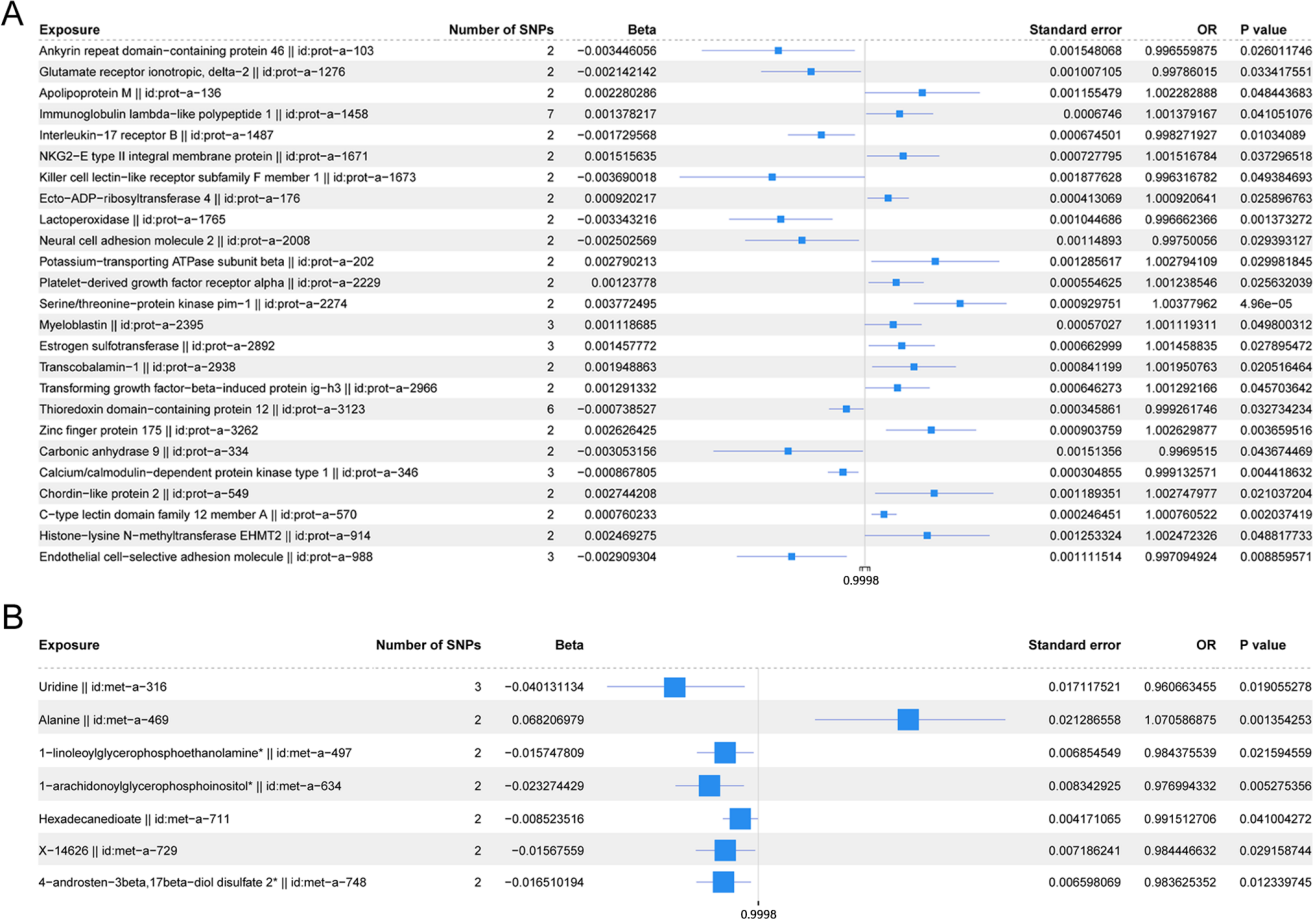
**(A)** Forest plot of the IVW model for plasma proteins on osteoporosis. **(B)** Forest plot of the IVW model for blood metabolites on osteoporosis.

**Figure 5** shows the scatter plot of the SNP effect estimates following MR analysis of plasma proteins in relation to osteoporosis (only the results with more than two SNPs are shown in the figure). The scatter plot fitting curves of the five models generally align in direction, with a relatively consistent slope across most models, and the intercept of the IVW model approaches zero. **Table 2** and **Figure 6A** show the results of the IVW model of plasma protein on osteoporosis. The IVW model results show that several plasma proteins exhibit a significant causal link with osteoporosis development (*P* < 0.05), including ankyrin repeat domain-containing protein 46 (ANKED46), glutamate receptor ionotropic delta-2, apolipoprotein M, immunoglobulin lambda-like polypeptide 1, interleukin-17 receptor B, integral NKG2-E type II protein, the killer cell lectin-like receptor subfamily F member 1 (KLRF1), ecto-ADP ribosyltransferase-4, lactoperoxidase (LPO), neural cell adhesion molecule 2, potassium-transporting ATPase subunit beta, platelet-derived growth factor receptor alpha, serine/threonine protein kinase pim-1, myeloblastin, estrogen sulfotransferase, transcobalamin-1, and transforming growth factor beta-induced protein ig-h3, thioredoxin domain-containing protein 12, zinc finger protein 175 (ZNF175), carbonic anhydrase 9 (CA9), and calcium/calmodulin-dependent protein kinase type 1, chordin-like protein 2, C-type lectin domain family 12 member A, histone lysine-N-methyltransferase EHMT2, and endothelial cell-selective adhesion molecule. Finally, we used the Steiger directionality test to validate the causal direction of plasma protein to osteoporosis (**Supplementary Table S11**). The Steiger directionally test calculated the variance explanation rate (*r* ^2^) of SNPs for exposure and outcome, respectively; the results showed that the variance explanation rate of SNPs for exposure was greater than for outcome, both directions were TRUE, and the *p*-values were less than 0.05, confirming the correct causal direction.

**Table 2:** MR causal effect estimation of plasma proteins and osteoporosis.

Exposure	ID	Number of SNPs	*β*	Standard error	OR (95%CI)	*p*-value
Ankyrin repeat domain-containing protein 46	prot-a-103	2	-0.00345	0.001548	0.996560 (0.993541, 0.999588)	0.026012
Glutamate receptor ionotropic, delta-2	prot-a-1276	2	-0.00214	0.001007	0.997860 (0.995892, 0.999832)	0.033418
Apolipoprotein M	prot-a-136	2	0.00228	0.001155	1.002283 (1.000016, 1.004555)	0.048444
Immunoglobulin lambda-like polypeptide 1	prot-a-1458	7	0.001378	0.000675	1.001379 (1.000056, 1.002704)	0.041051
Interleukin-17 receptor B	prot-a-1487	2	-0.00173	0.000675	0.998272 (0.996953, 0.999593)	0.010341
NKG2-E type II integral membrane protein	prot-a-1671	2	0.001516	0.000728	1.001517 (1.000089, 1.002946)	0.037297
Killer cell lectin-like receptor subfamily F member 1	prot-a-1673	2	-0.00369	0.001878	0.996317 (0.992657, 0.999990)	0.049385
Ecto-ADP-ribosyltransferase 4	prot-a-176	2	0.00092	0.000413	1.000921 (1.000111, 1.001731)	0.025897
Lactoperoxidase	prot-a-1765	2	-0.00334	0.001045	0.996662 (0.994624, 0.998705)	0.001373
Neural cell adhesion molecule 2	prot-a-2008	2	-0.0025	0.001149	0.997501 (0.995257, 0.999749)	0.029393
Potassium-transporting ATPase subunit beta	prot-a-202	2	0.00279	0.001286	1.002794 (1.000270, 1.005324)	0.029982
Platelet-derived growth factor receptor alpha	prot-a-2229	2	0.001238	0.000555	1.001239 (1.000151, 1.002328)	0.025632
Serine/threonine-protein kinase pim-1	prot-a-2274	2	0.003772	0.00093	1.003780 (1.001952, 1.005610)	0.00005
Myeloblastin	prot-a-2395	3	0.001119	0.00057	1.001119 (1.000001, 1.002239)	0.0498
Estrogen sulfotransferase	prot-a-2892	3	0.001458	0.000663	1.001459 (1.000158,1.002761)	0.027895
Transcobalamin-1	prot-a-2938	2	0.001949	0.000841	1.001951 (1.000300, 1.003604)	0.020516
Transforming growth factor-beta-induced protein ig-h3	prot-a-2966	2	0.001291	0.000646	1.001292 (1.000025, 1.002561)	0.045704
Thioredoxin domain-containing protein 12	prot-a-3123	6	-0.00074	0.000346	0.999262 (0.998585, 0.999939)	0.032734
Zinc finger protein 175	prot-a-3262	2	0.002626	0.000904	1.002630 (1.000855, 1.004407)	0.00366
Carbonic anhydrase 9	prot-a-334	2	-0.00305	0.001514	0.996952 (0.993998, 0.999913)	0.043674
Calcium/calmodulin-dependent protein kinase type 1	prot-a-346	3	-0.00087	0.000305	0.999133 (0.998536, 0.999730)	0.004419
Chordin-like protein 2	prot-a-549	2	0.002744	0.001189	1.002748 (1.000413, 1.005088)	0.021037
C-type lectin domain family 12 member A	prot-a-570	2	0.00076	0.000246	1.000761 (1.000277, 1.001244)	0.002037
Histone-lysine N-methyltransferase EHMT2	prot-a-914	2	0.002469	0.001253	1.002472 (1.000013, 1.004938)	0.048818
Endothelial-cell-selective adhesion molecule	prot-a-988	3	-0.00291	0.001112	0.997095 (0.994925, 0.999270)	0.00886

SNP, single-nucleotide polymorphism; OR, odds ratio; CI, confidence interval.

The scatter plots of the estimated effect of the SNPs screened following the MR analysis of blood metabolism and osteoporosis are shown in **Figure 5** (only the results of more than two SNPs are shown). All kinds of scatter diagram of the model fitting curves have the same direction, most of the models of slope are consistent, and the intercept of the IVW model is close to zero. As for blood metabolism, the results of the IVW model are shown in **Table 3** and **Figure 6B**. The IVW model results show that the metabolism of blood of uridine, alanine, 1-linoleoylglycerophosphoethanolamine, 1-arachidonoylglycerophosphoinositol, hexadecanedioate, X-14626, 4-androsten-3 beta, and 17 beta-diol disulfate 2 has a significant causal relationship with the pathogenesis of osteoporosis (*P* < 0.05). Finally, the Steiger directionality test was used to ascertain the causal direction of blood metabolism to osteoporosis (**Supplementary Table S12**). The Steiger directionality test calculated the variance explanation rate (*r* ^2^) of SNPs for exposure and outcome, respectively, and the results show that the variance for exposure is greater than for outcome, the direction is TRUE, and the *p*-values are less than 0.05, suggesting that the causal direction was correct.

**Table 3:** MR causal effect estimation of blood metabolism and osteoporosis.

Exposure	ID	Number of SNPs	*β*	Standard error	OR (95CI)	*p*-value
Uridine	met-a-316	3	-0.04013	0.017118	0.960663 (0.928968, 0.993441)	0.019055
Alanine	met-a-469	2	0.068207	0.021287	1.070587 (1.026839, 1.116198)	0.001354
1-Linoleoylglycerophosphoethanolamine	met-a-497	2	-0.01575	0.006855	0.984376 (0.971239, 0.997690)	0.021595
1-Arachidonoylglycerophosphoinositol	met-a-634	2	-0.02327	0.008343	0.976994 (0.961148, 0.993102)	0.005275
Hexadecanedioate	met-a-711	2	-0.00852	0.004171	0.991513 (0.983440, 0.999652)	0.041004
X-14626	met-a-729	2	-0.01568	0.007186	0.984447 (0.970678, 0.998411)	0.029159
4-Androsten-3beta,17beta-diol disulfate 2	met-a-748	2	-0.01651	0.006598	0.983625 (0.970987, 0.996428)	0.01234

SNP, single-nucleotide polymorphism; OR, odds ratio; CI, confidence interval.

### 3.4 Sensitivity analysis

#### 3.4.1 Obesity-related indicators

The Cochran Q test and *I* ^2^ statistic results (**Supplementary Table S13**) indicated that the heterogeneity of the MR results for most obesity-related indicators on osteoporosis was not significant (Cochran Q *p*-value >0.05) and the heterogeneity ratio was low (*I* ^2^ < 50%). The funnel plots of the instrumental variables of most obesity-related indicators are shown in **Supplementary Figures S1** and **S2**, which only show the results when the SNPs are more than two. The scatter points of causal association effects are essentially symmetrically distributed on both sides of the IVW model line, indicating that there is no potential bias in the results. Indicators with SNP numbers less than three could not be included for subsequent pleiotropic inspection and leave-one-out test. For a highly heterogeneous index, the IVW random-effects model was used to estimate the causal effect, with the results presented in **Table 4**.

**Table 4:** IVW random-effects model analysis of obesity-related indicators on osteoporosis.

Exposure	ID	Number of SNPs	*β*	Standard error	*P*-value
Waist circumference	ieu-a-61	39	-0.01053	0.002406	1.21E-05
Waist circumference	ieu-a-69	21	-0.00625	0.002952	0.034201
Body mass index	ieu-a-785	28	-0.00548	0.002323	0.018404
Waist-to-hip ratio	ieu-a-81	32	-0.00401	0.001999	0.044924
Body mass index	ieu-a-835	65	-0.00529	0.001835	0.003929
Body mass index	ieu-b-40	446	-0.0063	0.00111	1.38E-08
Body mass index	ukb-a-248	275	-0.0052	0.001089	1.78E-06
Leg fat percentage (right)	ukb-a-274	222	-0.00494	0.002146	0.021407
Arm fat percentage (right)	ukb-a-282	213	-0.005	0.001685	0.002994
Arm fat percentage (left)	ukb-a-286	231	-0.00515	0.001689	0.002307
Waist circumference	ukb-a-382	200	-0.00379	0.001578	0.01633
Arm fat percentage (right)	ukb-b-12854	338	-0.00546	0.001605	0.000661
Body mass index	ukb-b-19953	379	-0.00579	0.001134	3.20E-07
Arm fat percentage (left)	ukb-b-20188	342	-0.005	0.001609	0.001886
Body mass index	ukb-b-2303	374	-0.00559	0.00113	7.49E-07
Body fat percentage	ukb-b-8909	333	-0.00431	0.001709	0.011631
Waist circumference	ukb-b-9405	320	-0.00624	0.001504	3.32E-05

Based on the MR-Egger regression results, the *p*-values from the statistical hypothesis tests of the intercept terms for each index exceeded 0.05, and the intercept was close to zero. This implies that horizontal pleiotropy did not influence the causal inferences between obesity-related indicators and osteoporosis in this study (**Supplementary Table S14**).

The sensitivity analysis of the results using the leave-one-out test revealed no significant changes in the effect estimates for obesity-related measures, indicating the stability of the results (**Supplementary Table S15**, mr_leaveoneout_plot_obesity-related indicators.zip).

#### 3.4.2 Plasma protein

The Cochran Q test and *I* ^2^ statistic results (**Supplementary Table S16**) showed that the heterogeneity of the MR results of the selected plasma proteins related to osteoporosis was not significant (Cochran Q *p*-value >0.05) and the heterogeneity ratio was low (*I* ^2^ < 50%). The funnel plots of instrumental variables for most plasma proteins, as shown in **Supplementary Figure S3** (only presenting the results for SNPs with a count greater than two), demonstrated that the causal correlation effect of scatter in the IVW model distribution is symmetrical on each side, suggesting that the result does not have potential bias. Indicators with SNP counts less than three were not included for subsequent pleiotropic inspection and leave-one-out test.

According to the MR-Egger regression results, the *p*-values from the statistical hypothesis tests for the intercept terms of each index were greater than 0.05, and the intercept was close to zero. This suggests that horizontal pleiotropy did not influence the causal inferences between plasma proteins and osteoporosis (**Supplementary Table S17**).

The sensitivity analysis of the results using the leave-one-out test revealed no significant changes in the effect estimates for plasma proteins, indicating the stability of the results (**Supplementary Table S18**, mr_leaveoneout_plot_ plasma proteins.zip).

#### 3.4.3 Blood metabolites

Based on the Cochran Q test and *I* ^2^ statistic results (**Supplementary Table S19**), we observed that the MR analysis of most blood metabolites selected on osteoporosis was with no significant heterogeneity (Cochran Q *p*-value >0.05) or relatively low heterogeneity (*I* ^2^ < 50%). The funnel plot of the instrumental variables of blood metabolites is shown in **Supplementary Figure S4** (only shows the results when the number of SNPs is more than two), and the scatter of the causal association effect is essentially symmetrically distributed on both sides of the IVW model line, suggesting that there is no potential bias in the results. Indicators with fewer than three SNPs could not undergo subsequent horizontal pleiotropy test and leave-one-out test.

According to the MR-Egger regression results, the *p*-values from the statistical hypothesis tests of the intercept terms for each index were greater than 0.05, and the intercept was close to zero. This implies that horizontal pleiotropy did not influence the causal inferences between blood metabolites and osteoporosis (**Supplementary Table S20**).

The sensitivity analysis of the results using the leave-one-out test did not show significant changes in the effect estimates for blood metabolites, suggesting the stability of the results (**Supplementary Table S21**, mr_leaveoneout_plot_blood metabolism.zip).

### 3.5 Multivariate MR analysis

Based on the abovementioned results, we identified obesity-related indicators, plasma proteins, and blood metabolites that have a significant causal relationship with osteoporosis. We then conducted MR analysis of obesity-related indicators for plasma proteins and blood metabolites, respectively (**Tables 5**, **6**). Combinations with significant causality in the IVW model, positive Steiger directivity test results, and *p*-values less than 0.05 were selected and presented (**Supplementary Tables S22**, **S23**).

**Table 5:** MR causal effect estimation of obesity-related indicators and plasma proteins.

Exposure	Outcome	ID of outcome	Number of SNPs	*β*	Standard error	OR (95%CI)	*p*-value
Waist circumference							
ieu-a-103	Immunoglobulin lambda-like polypeptide 1	prot-a-1458	2	-0.72046	0.298758	0.486526 (0.270893, 0.873805)	0.015886
ieu-a-63	Apolipoprotein M	prot-a-136	16	-0.67468	0.201838	0.509321 (0.342912, 0.756484)	0.00083
ieu-a-63	Ecto-ADP-ribosyltransferase 4	prot-a-176	16	-0.4155	0.201718	0.660013 (0.444475, 0.980073)	0.039419
ieu-a-63	Neural cell adhesion molecule 2	prot-a-2008	16	-0.54038	0.201728	0.582530 (0.392287, 0.865032)	0.00739
ieu-a-69	Neural cell adhesion molecule 2	prot-a-2008	21	-0.36446	0.178406	0.694569 (0.489613, 0.985321)	0.041063
ieu-a-69	Histone-lysine N-methyltransferase EHMT2	prot-a-914	21	-0.43035	0.169208	0.650279 (0.466731, 0.906010)	0.01098
ieu-a-71	Platelet-derived growth factor receptor alpha	prot-a-2229	25	0.378946	0.156648	1.460745 (1.074566, 1.985708)	0.015559
ieu-a-63	Histone-lysine N-methyltransferase EHMT2	prot-a-914	16	-0.40542	0.201746	0.666698 (0.448951, 0.990054)	0.044479
Waist-to-hip ratio|							
ieu-a-109	Calcium/calmodulin-dependent protein kinase type 1	prot-a-346	5	0.561447	0.248887	1.753208 (1.076408, 2.855552)	0.024081
ieu-a-111	Glutamate receptor ionotropic, delta-2	prot-a-1276	7	0.410209	0.203416	1.507133 (1.011580, 2.245448)	0.043737
ieu-a-111	Thioredoxin domain-containing protein 12	prot-a-3123	7	-0.423	0.203338	0.655077 (0.439752, 0.975837)	0.037499
ieu-a-111	Calcium/calmodulin-dependent protein kinase type 1	prot-a-346	7	0.509834	0.221901	1.665015 (1.077785, 2.572198)	0.021586
ieu-a-75	Thioredoxin domain-containing protein 12	prot-a-3123	22	-0.52253	0.160226	0.593017 (0.433192, 0.811810)	0.001109
ieu-a-75	Zinc finger protein 175	prot-a-3262	22	0.367649	0.160295	1.444335 (1.054926, 1.977489)	0.021815
ieu-a-75	Calcium/calmodulin-dependent protein kinase type 1	prot-a-346	22	0.51394	0.160266	1.671865 (1.221180, 2.288878)	0.001342
ieu-a-75	Histone-lysine N-methyltransferase EHMT2	prot-a-914	22	-0.42633	0.160231	0.652900 (0.476931, 0.893796)	0.007797
ieu-a-81	Calcium/calmodulin-dependent protein kinase type 1	prot-a-346	32	0.37042	0.129696	1.448343 (1.123237, 1.867546)	0.004289
ieu-a-81	Histone-lysine N-methyltransferase EHMT2	prot-a-914	32	-0.3624	0.129708	0.696005 (0.539762, 0.897475)	0.005207
Body mass index							
ieu-a-95	Neural cell adhesion molecule 2	prot-a-2008	7	-0.50492	0.240349	0.603556 (0.376815, 0.966734)	0.035662
ieu-a-974	Neural cell adhesion molecule 2	prot-a-2008	35	-0.26738	0.129466	0.765383 (0.593848, 0.986466)	0.038899

SNP, single-nucleotide polymorphism.

**Table 6:** MR causal effect estimation of obesity-related indicators and blood metabolites.

Exposure	Outcome	ID of outcome	Number of SNPs	*β*	Standard error	OR (95%CI)	*p*-value
Waist circumference							
ieu-a-103	1-Arachidonoylglycerophosphoinositol	met-a-634	2	0.074233	0.031857	1.077058 (1.011863, 1.146454)	0.019795
ieu-a-105	Alanine	met-a-469	4	0.103511	0.033395	1.109058 (1.038790, 1.184079)	0.001938
ieu-a-63	1-Linoleoylglycerophosphoethanolamine	met-a-497	16	-0.069575	0.0288	0.932791 (0.881596, 0.986959)	0.0157
ieu-a-65	Uridine	met-a-316	13	-0.026681	0.012655	0.973672 (0.949818, 0.998124)	0.035001
ukb-a-382	1-Linoleoylglycerophosphoethanolamine	met-a-497	155	-0.035158	0.01733	0.965452 (0.933209, 0.998810)	0.042488
Body mass index							
ebi-a-GCST006368	1-Linoleoylglycerophosphoethanolaminei	met-a-497	134	-0.044739	0.015269	0.956247 (0.928053, 0.985297)	0.003389
ieu-a-835	1-Linoleoylglycerophosphoethanolamine	met-a-497	65	-0.045057	0.018059	0.955943 (0.922698, 0.990385)	0.012597
ieu-a-94	1-Arachidonoylglycerophosphoinositol	met-a-634	7	0.064025	0.021039	1.066119 (1.023051, 1.111000)	0.002341
ieu-a-95	1-Linoleoylglycerophosphoethanolamine	met-a-497	7	-0.058893	0.029474	0.942808 (0.889887, 0.998876)	0.045698
ieu-a-974	1-Arachidonoylglycerophosphoinositol	met-a-634	35	0.031895	0.014187	1.032409 (1.004097, 1.061521)	0.024565
Leg fat percentage (left)							
ukb-a-278	1-Linoleoylglycerophosphoethanolamine	met-a-497	171	-0.050609	0.023948	0.950650 (0.907059, 0.996336)	0.034579
Arm fat percentage (right)							
ukb-a-282	1-Linoleoylglycerophosphoethanolamine	met-a-497	161	-0.074039	0.019572	0.928635 (0.893687, 0.964950)	0.000155
Arm fat percentage (left)							
ukb-a-286	1-Linoleoylglycerophosphoethanolamine	met-a-497	178	-0.055483	0.018898	0.946028 (0.911627, 0.981727)	0.003326
Body fat percentage							
ukb-b-8909	Uridine	met-a-316	263	-0.021271	0.008072	0.978954 (0.963587, 0.994565)	0.008409

SNP, single-nucleotide polymorphism.

Taking these significant results as exposure, we conducted a separate MVMR analysis for osteoporosis, excluding the combinations that could not be used for MVMR analysis. We obtained 20 significant MVMR models for the effect of plasma protein-mediated obesity-related indicators on osteoporosis (**Table 7**). The results show that the relationship between plasma proteins and osteoporosis in model 11 is significant (*P* < 0.05), while the relationships in the remaining models are not significant (*P* > 0.05). For the effect of blood-metabolite-mediated obesity-related indicators on osteoporosis, we identified 14 meaningful MVMR models (**Table 8**). The results show that the relationship between blood metabolites and osteoporosis in model 23 is significant (*P* < 0.05), while the relationships in the other models are not significant (*P* > 0.05).

**Table 7:** Results of MVMR analysis of the effect of plasma proteins and obesity-related indicators on osteoporosis.

Model	Exposure	ID	*β*	Standard error	*p*-value
Model 1	Waist circumference	ieu-a-103	-0.01773	NA	NA
	Immunoglobulin lambda-like polypeptide 1	prot-a-1458	-0.01155	NA	NA
Model 2	Waist-to-hip ratio	ieu-a-109	-0.01011	0.006198	0.102725
	Calcium/calmodulin-dependent protein kinase type 1	prot-a-346	-0.00755	0.009613	0.432177
Model 3	Waist-to-hip ratio	ieu-a-111	-0.0071	0.003364	0.034691
	Glutamate receptor ionotropic	prot-a-1276	-0.00643	0.006686	0.336094
Model 4	Waist-to-hip ratio	ieu-a-111	-0.00453	0.004108	0.269684
	Thioredoxin domain-containing protein 12	prot-a-3123	0.00916	0.006605	0.165503
Model 5	Waist-to-hip ratio	ieu-a-111	-0.00652	0.003245	0.044488
	Calcium/calmodulin-dependent protein kinase type 1	prot-a-346	-0.00613	0.00474	0.195866
Model 6	Waist circumference	ieu-a-63	-0.01054	0.0043	0.014216
	Apolipoprotein M	prot-a-136	0.000788	0.005119	0.877664
Model 7	Waist circumference	ieu-a-63	-0.01081	0.003477	0.001879
	Ecto-ADP-ribosyltransferase 4	prot-a-176	0.000514	0.004309	0.90499
Model 8	Waist circumference	ieu-a-63	-0.00844	0.003609	0.0194
	Neural cell adhesion molecule 2	prot-a-2008	0.005002	0.004176	0.230985
Model 9	Waist circumference	ieu-a-63	-0.01171	0.003441	0.000666
	Histone-lysine N-methyltransferase EHMT2	prot-a-914	-0.00175	0.004139	0.672618
Model 10	Waist circumference	ieu-a-69	-0.00644	0.003164	0.04177
	Neural cell adhesion molecule 2	prot-a-2008	-0.00061	0.003676	0.867845
Model 11	Waist circumference	ieu-a-69	-0.00162	0.003532	0.646788
	Histone-lysine N-methyltransferase EHMT2	prot-a-914	0.009651	0.004732	0.041386
Model 12	Waist circumference	ieu-a-71	0.004514	0.002965	0.127881
	Platelet-derived growth factor receptor alpha	prot-a-2229	0.000742	0.004276	0.862166
Model 13	Waist-to-hip ratio	ieu-a-75	-0.00387	0.002929	0.185953
	Thioredoxin domain-containing protein 12	prot-a-3123	0.006199	0.00344	0.071556
Model 14	Waist-to-hip ratio	ieu-a-75	-0.00648	0.002879	0.024355
	Zinc finger protein 175	prot-a-3262	-0.00105	0.003654	0.773264
Model 15	Waist-to-hip ratio	ieu-a-75	-0.00499	0.002942	0.089579
	Calcium/calmodulin-dependent protein kinase type 1	prot-a-346	-0.00399	0.003296	0.225949
Model 16	Waist-to-hip ratio	ieu-a-75	-0.00682	0.002984	0.022189
	Histone-lysine N-methyltransferase EHMT2	prot-a-914	3.76E-05	0.003799	0.992109
Model 17	Waist-to-hip ratio	ieu-a-81	-0.00316	0.002147	0.141242
	Calcium/calmodulin-dependent protein kinase type 1	prot-a-346	-0.0018	0.002729	0.510748
Model 18	Waist-to-hip ratio	ieu-a-81	-0.00351	0.002193	0.109641
	Histone-lysine N-methyltransferase EHMT2	prot-a-914	0.001943	0.002877	0.499457
Model 19	Body mass index	ieu-a-95	-0.0029	0.003119	0.352809
	Neural cell adhesion molecule 2	prot-a-2008	0.00483	0.004559	0.289434
Model 20	Body mass index	ieu-a-974	-0.00575	0.001947	0.003133
	Neural cell adhesion molecule 2	prot-a-2008	0.001098	0.002604	0.673261

SNP, single-nucleotide polymorphism; *β*, effect coefficients in multivariate Mendelian randomization analysis.

**Table 8:** Results of MVMR analysis of the effects of blood metabolites and obesity-related indicators on osteoporosis.

Model	Exposure	ID	*β*	Standard error	*p*-value
Model 21	Body mass index	ebi-a-GCST006368	-0.00677	0.001539	1.08E-05
	1-Linoleoylglycerophosphoethanolamine	met-a-497	-0.00589	0.008775	0.502016
Model 22	Waist circumference	ieu-a-103	-0.00946	0.002879	0.001009
	1-Arachidonoylglycerophosphoinositol	met-a-634	-0.00708	0.009608	0.461249
Model 23	Waist circumference	ieu-a-105	-0.01405	0.007309	0.054577
	Alanine	met-a-469	0.072537	0.028385	0.010605
Model 24	Waist circumference	ieu-a-63	-0.01121	0.002913	0.00012
	1-Linoleoylglycerophosphoethanolamine	met-a-497	-0.00263	0.010645	0.804847
Model 25	Waist circumference	ieu-a-65	-0.00846	0.002453	0.000567
	Uridine	met-a-316	-0.04118	0.025068	0.100443
Model 26	Body mass index	ieu-a-835	-0.00559	0.001841	0.002414
	1-Linoleoylglycerophosphoethanolamine	met-a-497	-0.00938	0.007869	0.233207
Model 27	Body mass index	ieu-a-94	-0.00432	0.002908	0.136928
	1-Arachidonoylglycerophosphoinositol	met-a-634	-0.01542	0.017239	0.371197
Model 28	Body mass index	ieu-a-95	-0.00526	0.002385	0.027516
	1-Linoleoylglycerophosphoethanolamine	met-a-497	-0.00569	0.009623	0.554518
Model 29	Body mass index	ieu-a-974	-0.0059	0.001986	0.002986
	1-Arachidonoylglycerophosphoinositol	met-a-634	-0.0004	0.014114	0.9776
Model 30	1-Linoleoylglycerophosphoethanolamine	met-a-497	0.000871	0.010186	0.93184
	Leg fat percentage (left)	ukb-a-278	-0.0184	0.004728	9.98E-05
Model 31	1-Linoleoylglycerophosphoethanolamine	met-a-497	0.000527	0.010779	0.961031
	Arm fat percentage (right)	ukb-a-282	-0.01499	0.003456	1.45E-05
Model 32	1-Linoleoylglycerophosphoethanolamine	met-a-497	9.92E-06	0.010356	0.999235
	Arm fat percentage (left)	ukb-a-286	-0.01412	0.003298	1.87E-05
Model 33	1-Linoleoylglycerophosphoethanolamine	met-a-497	-0.01287	0.00965	0.182195
	Waist circumference	ukb-a-382	-0.01174	0.003173	0.000216
Model 34	Uridine	met-a-316	0.002116	0.040166	0.957984
	Body fat percentage	ukb-b-8909	-0.01406	0.003662	0.000123

SNP, single-nucleotide polymorphism; *β*, effect coefficients in multivariate Mendelian randomization analysis.

### 3.6 Mediation effect analysis

In the MVMR analysis, models demonstrating a significant causal relationship between mediating factors and outcomes were evaluated for mediating effects. The Sobel test was employed to determine whether the mediating effects were significant for the remaining models, and the mediating effects were evaluated for those models that showed significance. Among the effect models of obesity-related indicators mediated by plasma proteins on osteoporosis, only model 11 showed significant effects between plasma protein and osteoporosis (*P* < 0.05), while the Sobel test results (**Supplementary Table S24**) indicated no significant mediating effects in the remaining models (*P* < 0.05). Therefore, we only discuss the possible mediating effects in model 11. Since there was no significant causal relationship between obesity-related indicators and osteoporosis in model 11 (*P* < 0.05), model 11 might represent a case of complete mediation, and the results are shown in **Table 9**. Among the effect models of obesity-related indicators mediated by blood metabolites on osteoporosis, only model 23 showed significant effects between blood metabolites and osteoporosis (*P* < 0.05), while the Sobel test results (**Supplementary Table S25**) show no significant mediating effects in the remaining models (*P* < 0.05). Therefore, we only discuss the possible mediating effects in model 23. Given the absence of a causal relationship between obesity-related indicators and osteoporosis in model 23 (*P* < 0.05), model 23 may represent a case of complete mediation, and the results are shown in **Table 9**.

**Table 9:** Effect of plasma proteins and blood metabolites mediating obesity-related indicators on osteoporosis through MR.

Model	Exposure (E)	Mediator (M)	Direct effect E–M (95% CI)	Direct effect M–O (95% CI)	Mediation effect (95% CI)	Direct effect E–O	Total effect E–O
Model 11	Waist circumference (id: ieu-a-69)	Histone-lysine N-methyltransferase EHMT2 (id: prot-a-914)	-0.430353 (-0.762002, -0.098705)	0.009651 (0.000377, 0.018924)	-0.004153 (-0.004222, -0.004085)	-0.001618 (-0.008541, 0.005304)	-0.006252 (-0.012038, -0.000466)
Model 23	Waist circumference (id: ieu-a-105)	Alanine (id: met-a-469)	0.103511 (0.038057, 0.168965)	0.072537 (0.016902, 0.128172)	0.007508 (0.006208, 0.008809)	-0.014050 (-0.028376, 0.000276)	-0.012115 (-0.024228, -0.000001)

CI, confidence interval.

## 4 Discussion

It is reported that osteoporosis has become a major global health problem. Historically, obesity was considered protective against osteoporosis (30). Recently, the study of biomarkers related to osteoporosis in proteomics and metabolomics has attracted many researchers (31, 32). We performed the first comprehensive two-sample MR analysis to evaluate the causal relationships and potential mediating factors between obesity-related indicators and osteoporosis. The results indicate that 31 obesity-related indicators may have a causal relationship with osteoporosis. Most of the obesity-related indicators, including BMI, waist circumference, waist-to-hip ratio, fat percentage, arm fat percentage, and body fat percentage, may reduce the risk of osteoporosis. There may be a causal relationship between 25 plasma protein markers and osteoporosis, among which serine/threonine-protein kinase pim-1, ATP1B1, ZFP175, and chordin-like protein 2 may significantly increase the risk of osteoporosis, while ANKED46, KLRF1, LPO, and CA9 may significantly reduce the risk of osteoporosis. There may be a causal relationship between seven blood metabolite markers and osteoporosis. Alanine may increase the risk of osteoporosis, while uridine and 1-linoleoylglycerophosphoethanolamine may lower the risk of osteoporosis. The causal relationship between obesity-related indexes, plasma protein, blood metabolites, and osteoporosis was confirmed. In the sensitivity analysis, the heterogeneity of obesity-related indicators, plasma proteins, and blood metabolites was not significant and was not affected by horizontal pleiotropy, indicating stability. In the MVMR analysis, the reduced risk of osteoporosis caused by obesity-related indicators may be mediated by EHMT2 among plasma proteins and alanine among blood metabolites.

Previous studies have shown that obesity could prevent osteoporosis (33). It is reported that a low BMI is considered as an important risk factor for osteoporosis (34, 35). A cross-sectional study involving 3,774 men over 50 and 4,982 postmenopausal women found that when BMI increased by 1 kg/m^2^, men and women reduced their risk of osteoporosis by 28% and 13%, respectively (36). In our MR study, the genetic prediction of BMI is closely related to osteoporosis. The increase in BMI could reduce the risk of osteoporosis, which is consistent with the results of previous studies. Our research shows that waist circumference (id: ieu-a-71) could increase the risk of osteoporosis, while an increase in waist circumference from other data sources might reduce the risk of osteoporosis. This discrepancy may be due to different data sources employing various research designs, data collection tools, or analytical methods, and these differences might lead to variations in the direction of the effect curve. Additionally, the inconsistency in the direction of the effect estimation curve might also be attributed to incidental factors. In cases of small samples or large data noise, random errors might lead to instability in effect estimation results, resulting in the inconsistent direction of curves across different data sources. Waist circumference (id: ieu-a-71) is derived from the GWAS database and comprises a total of 104,079 samples, which might account for some differences. According to a research report, there is a significant correlation between waist circumference and BMD (37). Tian H et al. (38) included a cohort study of 8,475 subjects and found that waist circumference was negatively correlated with the risk of osteoporosis. Zheng S et al. also believe that a higher obesity index, including waist circumference and waist-to-hip ratio, could significantly reduce the risk of osteoporosis (39). Some scholars argue that fat content has a positive effect on BMD in women (40), which is consistent with our genetic prediction study.

The results of the IVW model showed that 15 plasma protein markers may increase the risk of osteoporosis, and there may be a strong causal relationship between serine/threonine-protein kinase PIM-1, potassium-transporting ATPase subunit beta (ATP1B1), ZFP175, chordin-like protein 2, and osteoporosis. The primary function of PIM kinase is to phosphorylate the serine/threonine residues of target proteins, which can be divided into three types: PIM-1, PIM-2 and PIM-3. PIM-1 has functions in regulating cell growth, differentiation, cell cycle, senescence, and apoptosis (41, 42). The expression of ATP1B1 can inhibit virus replication and increase the levels of IFNs, IFN-stimulating genes, and inflammatory cytokines (43). Zinc finger proteins (ZFP) constitute a large and heterogeneous protein family distinguished by the presence of one or more zinc finger domains, where zinc is crucial for maintaining structural stability. These proteins have the ability to interact with DNA, RNA, lipids, and other proteins, thereby participating in diverse cellular functions such as transcriptional control, mRNA degradation, ubiquitin-dependent protein degradation, and mRNA stabilization (44). Bone morphogenetic protein (BMP) is generally considered to induce stem cells to differentiate into osteoblasts. Chordin-like protein is a secreted protein that regulates the expression and function of BMP. Some scholars have found that chordin-like protein can enhance the role of BMP in inducing osteoblast differentiation (45, 46). A total of 10 plasma protein markers could reduce the risk of osteoporosis, among which ANKED46, killer cell lectin-like receptor subfamily F member 1 (KLRF1), LPO, and CA9 may have a strong causal relationship with osteoporosis. It is reported that ANKED46 encodes an anchor protein repeat domain 46, which plays a role in protein regulation, apoptosis, cell adhesion and migration, and cell proliferation. Increasing ANKED46 could inhibit cell proliferation, cell migration, and tumor growth (47). Yang YJ et al. found that ANKED46 has a strong correlation with bone remodeling and may be an important target for exercise-based interventions aimed at enhancing bone mass and combating postmenopausal osteoporosis (48). Initially identified as a component of human cDNA, KLRF1 shows homology to human NKRP1A in the expressed sequence tag database. When activated in NK cells, KLRF1 receptors trigger calcium mobilization and cytotoxicity. Additionally, KLRF1 serves as an indicator of NK cell maturation within secondary lymphoid tissue (49–52). LPO is a heme peroxidase that inhibits osteoclast formation by inhibiting RANKL/RANK signal transduction (53). CA9 is a transmembrane zinc metalloprotein that catalyzes a very basic but vital physiological reaction: the conversion of carbon dioxide to bicarbonate by the release of protons (54). CA9 is a direct target of hypoxia-inducible factor (HIF) and could be used as an alternative marker and prognostic indicator of hypoxia. The inhibition of carbonic anhydrase may be related to the treatment of osteoporosis (55).

In recent years, metabolomics has become a focus point in disease mechanism research. Metabolomics can provide a deeper understanding of the biological mechanism of disease by identifying altered metabolites or metabolic pathways. Chun LF et al. believe that children with lower BMD have higher levels of alanine aminotransferase (56), Panahi N et al. believe that alanine levels in women are negatively correlated with osteoporosis (57), while our predictive model found that alanine could increase the risk of osteoporosis, which might be attributed to differences in race, age, and sex. The IVW model shows that six blood metabolites could reduce the risk of osteoporosis, among which uridine and 1-linoleoylglycerophosphoethanolamine may have a strong causal relationship with osteoporosis. Uridine is a type of nucleotide that significantly impacts the synthesis of RNA, glycogen, and biofilm (58). Studies have found that uridine has antioxidant stress effects and could delay the senescence of chondrocytes and mesenchymal stem cells *in vivo (* 59). Recently, MR has been widely used in the study of disease etiology. A MR study found that 1-linoleoylglycerophosphoethanolamine is a high-risk blood metabolite for lacunar stroke (60). Another MR study shows that 1-linoleoylglycerophosphoethanolamine directly affects colorectal cancer, independent of other metabolites, and has a protective effect on colorectal cancer (61). 1-Linoleoylglycerophosphoethanolamine is an important component of phospholipid ethanolamine. As a main component of cell membrane phospholipids, phospholipid ethanolamine plays a crucial role in maintaining the stability of cell fine structure. Currently, there is no research on the relationship between 1-linoleoylglycerophosphoethanolamine and osteoporosis. Our gene prediction study found that 1-linoleoylglycerophosphoethanolamine may reduce the risk of osteoporosis, which might provide a foundation for subsequent genetic research of osteoporosis and draw scholars’ attention to the relationship between metabonomic and osteoporosis.

Further MVMR and mediating MR found that the plasma protein EHMT2 and blood metabolite alanine may mediate the effects of obesity-related indicators, specifically waist circumference, on osteoporosis. This suggests that obesity may exert a protective effect on osteoporosis through the mediation of EHMT2 and alanine.

EHMT2 (G9a) is a euchromatin-localized histone methyltransferase playing a crucial role in epigenetic regulation, and it mediates the methylation of histone H3 at lysines 9 and 27 (H3K9 and H3K27) (62). Both obesity and osteoporosis are influenced by genetically determined factors, with adipocytes and osteoblasts originating from the common bone marrow mesenchymal stem cell (BMSC). This suggests that pleiotropic genes regulate these two processes. By affecting the expression of these pleiotropic genes, EHMT2 may indirectly influence the onset of obesity and osteoporosis. In the epigenetic regulation of obesity-related genes, G9a modulates their transcriptional activity by binding to the promoter regions of these genes, thereby influencing fat metabolism. For instance, G9a regulates myosin levels in muscle tissue, affecting muscle function, which may indirectly impact energy expenditure and fat accumulation (63). The link between G9a and metabolic diseases was recently reported. In hepatocytes, G9a regulates the development of metabolic diseases, including obesity and insulin resistance, through the regulation of HMGA1 (64). Furthermore, G9a may regulate the differentiation and function of adipocytes by influencing the insulin signaling pathway, thereby affecting whole-body fat distribution and metabolism. It has been shown that G9a plays a role in MMP-9-dependent H3NT protein hydrolysis and gene transcription during RANKL-induced osteoclast differentiation by catalyzing H3K27me1 (65). G9a also has a direct effect on the expression of osteoclastogenic genes (66). G9a exerts an inhibitory effect on osteoclastogenesis by regulating NFATc1 function, thereby influencing the process of bone resorption (67). Therefore, inhibition of G9a might protect against osteoporosis by reducing osteoclastogenesis and increasing bone density. The results of our study also support the idea that obesity might protect against osteoporosis by inhibiting G9a.

Amino acids, peptides, and their derivatives are frequently identified as key metabolites associated with BMD in metabolomics studies. These compounds are often among those disrupted in osteoporosis, thereby significantly impacting bone health (68). They modulate bone remodeling through various mechanisms, such as stimulating osteoblast proliferation and differentiation, enhancing collagen production, and acting as signaling molecules to control bone turnover (69). Our findings suggest that the blood metabolite alanine may mediate the protective effect of obesity against osteoporosis and that alanine exhibits a positive correlation with osteoporosis.

A cross-sectional investigation revealed that identical twins with higher intakes of alanine and glycine exhibited notably higher spinal BMD compared to their counterparts. A significant positive correlation was observed between the intake of six bone-fortifying amino acids (alanine, arginine, glutamate, leucine, lysine, and proline) and BMD at both the spine and forearms (70). However, despite identifying alanine and other amino acids as beneficial for bone health, this cross-sectional study cannot establish a definitive causal link between alanine intake and BMD. A prospective cohort study identified valine, leucine, isoleucine, and alanine as the most important amino acids negatively associated with osteoporosis in women (OR: 0.77–0.89) (57). The results of this study are not consistent with ours. This study was a prospective cohort study with a small population and many confounding factors, which could not determine the causal relationship between alanine and osteoporosis. Moreover, the results of this study could only show the correlation between amino acids and osteoporosis and therefore might not be in agreement with the findings of our MR analysis. Additionally, a cross-sectional study of 103 spinal cord injury patients found that a higher alanine intake was not associated with BMD after controlling for confounding factors, including demographic and injury-related characteristics and calcium intake (71). The results of a recent MR study on amino acids and bone density do not support a causal relationship between alanine and bone density (72). Therefore, the relationship between alanine and BMD in previous studies seems to be unclear. Our study, however, primarily demonstrated that alanine may have a direct mediating effect on the protective effect of obesity against osteoporosis.

Obesity is associated with varying degrees of metabolic disorders, including protein metabolism. Altered regulation of protein metabolism in obese patients leads to reduced inhibition of systemic protein hydrolysis and normal or low-stimulation insulin and amino acid, thereby affecting bone metabolism (57, 73). Currently, there is no direct research validating the specific mechanisms through which alanine influences bone metabolism. Considering alanine’s roles in metabolic processes, including its involvement in protein synthesis, conversion to pyruvate for gluconeogenesis, and participation in glutathione synthesis affecting cellular antioxidant function, alanine may impact bone metabolism through several potential pathways (74, 75). As an activator of the mTORC1 signaling pathway, alanine could influence bone metabolism by modulating insulin signaling (76). IGF-1 promotes the proliferation and differentiation of osteoblasts, and alanine may affect osteoblast function by stimulating IGF-1 secretion (77). Additionally, alanine might indirectly regulate osteoclast activity by influencing inflammation and insulin signaling. Another possibility is that alanine could regulate bone cell differentiation by affecting the MAPK signaling pathway (78). However, further basic research is needed to elucidate the detailed mechanisms of alanine’s specific role in bone health.

In summary, our MR analysis, particularly the IVW model, revealed a significant causal association between obesity-related indicators and osteoporosis. This may provide preliminary evidence for the potential of obesity to contribute to osteoporosis. However, we should also notice that the observed odds ratios (ORs) were close to 1, suggesting that the effect size of obesity-related indicators on osteoporosis is relatively minor. This indicates the need to carefully assess the clinical significance and practical application value of the effect size, which remains to be validated. Future research should further verify these findings within a broader population and include comprehensive analyses with additional biomarkers to better understand the intricate relationship between obesity and osteoporosis. Our Steiger’s directionality tests support the causal effect direction from obesity-related indicators to osteoporosis, with the variance explained by the SNPs in both the exposure and the outcome aligning with expectations. Nonetheless, this does not rule out the potential influence of other confounding factors or biological mechanisms. Therefore, we advise caution when applying these findings in clinical practice and public health policy, especially in intervention development, as a thorough consideration of a wider range of risk factors and biological mechanisms is essential.

## 5 Limitations

Although this study employed a two-sample MR to investigate the causal relationships between obesity-related indicators, plasma proteins, blood metabolites, and osteoporosis, it still has several limitations. Firstly, the GWAS data used in this research was primarily derived from individuals of European descent. While this restricted selection helps to reduce the interference from ethnic differences, it also inherently limits the generalizability of our findings to other ethnic groups. In other words, these results may not be entirely applicable to populations with different genetic backgrounds or environmental factors. Moreover, due to the absence of detailed stratified analysis and validation across diverse populations, the global applicability of our conclusions may be somewhat limited. Future research should aim to validate these findings in various ethnicities and populations to further enhance the broad applicability and credibility of our study’s results.

Secondly, although we conducted sensitivity analyses to minimize bias, it is unlikely that bias can be entirely eliminated due to various reasons. In the selection of instrumental variables, we strictly adhered to the relevance assumption, ensuring that the chosen SNPs had significant correlations with the exposure factors. We set a *p*-value threshold of *p* < 5×10^-8^, a criterion widely recognized in the relevant literature (79). Furthermore, we assessed the validity of the instrumental variables by calculating the F-test statistics, ensuring that the selected SNPs were strong instrumental variables, thereby reducing the risk of bias introduced by weak instrumental variables. The F-test statistics were all greater than 10, indicating the rigor of our selection process and the limited potential for bias. Nonetheless, when using multiple SNPs for calculations, particularly in multiple analyses, there remains a risk of introducing false-positive results. Regarding the independence assumption, SNPs in linkage disequilibrium were excluded during the selection of instrumental variables, further reducing the risk of correlation with potential confounding factors that may affect either the exposure or the outcome. Although this method cannot completely eliminate all potential confounding factors, especially in complex biological processes, it significantly enhances the credibility of the analysis results. Concerning the exclusion restriction assumption, where SNPs are assumed to affect the outcome only through the specified path, while this was thoroughly considered during the selection of instrumental variables, some SNPs may still influence the outcome through other unmeasured pathways. Although we assessed pleiotropy using methods such as the MR-Egger test, we cannot fully exclude the possibility of bias arising from some SNPs simultaneously affecting both the exposure and the outcome through other unmeasured pathways. In future research, more advanced methods can be employed to bolster the reliability of the findings, such as Cholesky Decomposition-based Adjusted Variation Inflation Factor for Annotation of Rare variant Associations (CAVIAR) and Linkage Disequilibrium Score Chisq regression (LDSC). Additionally, future research should conduct validations in a more diverse population to ascertain the broader applicability of the identified causal associations.

## 6 Conclusion

The findings of this study suggest that obesity may have a certain protective effect against osteoporosis, potentially mediated by EHMT2 in plasma proteins and alanine in blood metabolites. The modulation of these factors could potentially aid in the prevention of osteoporosis. However, caution is advised when interpreting these findings. The relationship between obesity and osteoporosis is complex, with observational studies often yielding conflicting outcomes due to confounding factors. Moreover, MR studies have certain limitations and may not account for all relevant biological and environmental variables. Although our study has identified a statistical association, further empirical validation is necessary to fully comprehend the mechanisms underlying the impact of obesity on osteoporosis.

## Data Availability

Publicly available datasets were analyzed in this study. This data can be found here: MRC IEU OpenGWAS.
