# Supplementary figures and images for: Causal inference study of plasma proteins and blood metabolites mediating the effect of obesity-related indicators on osteoporosis
Source: Front Endocrinol (Lausanne). 2025 Feb 18;16:1435295. doi: 10.3389/fendo.2025.1435295 (PMC11876022; doi:10.3389/fendo.2025.1435295)

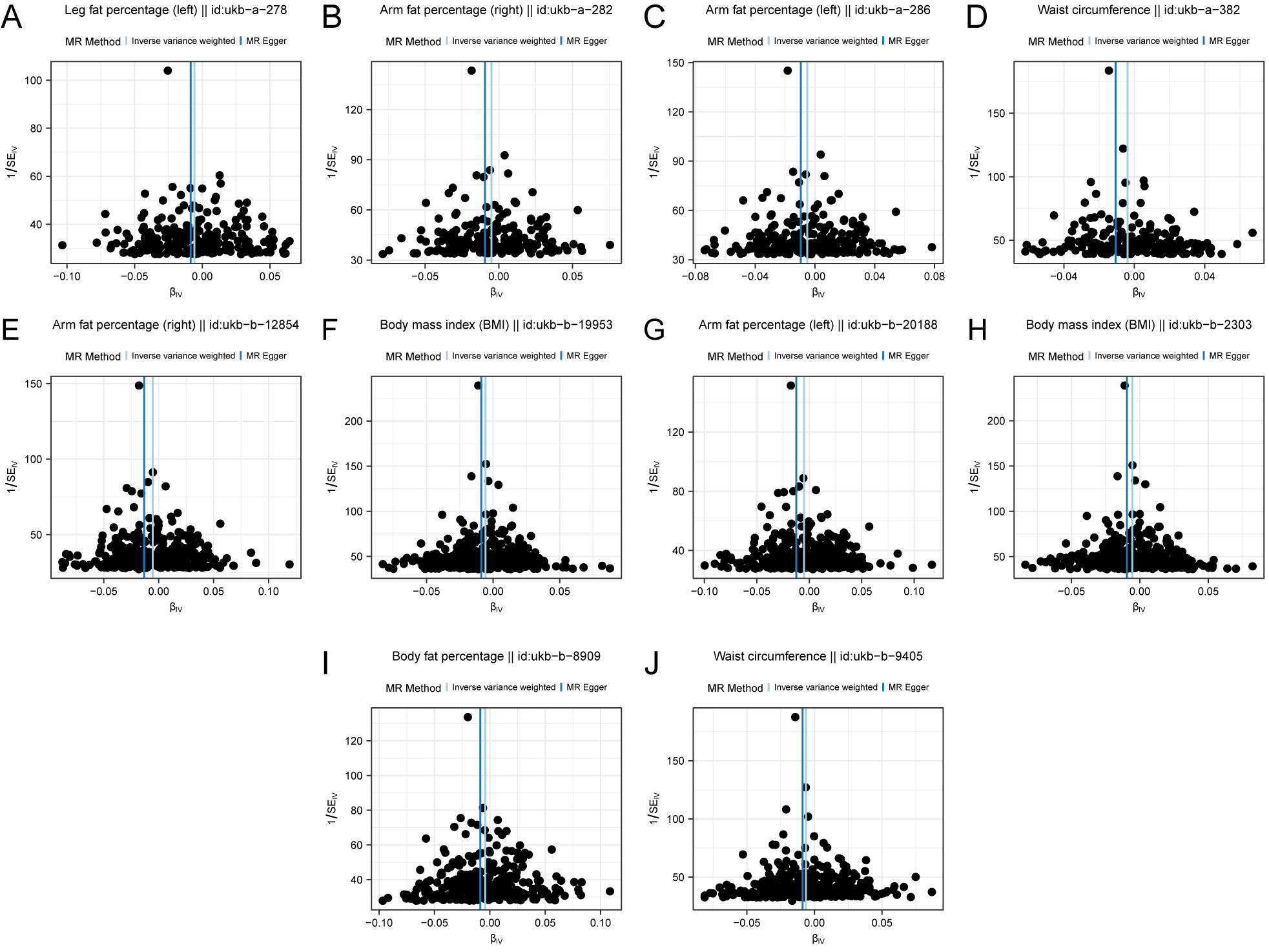

Supplement: Supplementary file 1 [file DataSheet1.zip › Supplementary Figures/Figure S1 Funnel plot of heterogeneity test for MR of obesity-related indicators (part 1) on osteoporosis .tif]

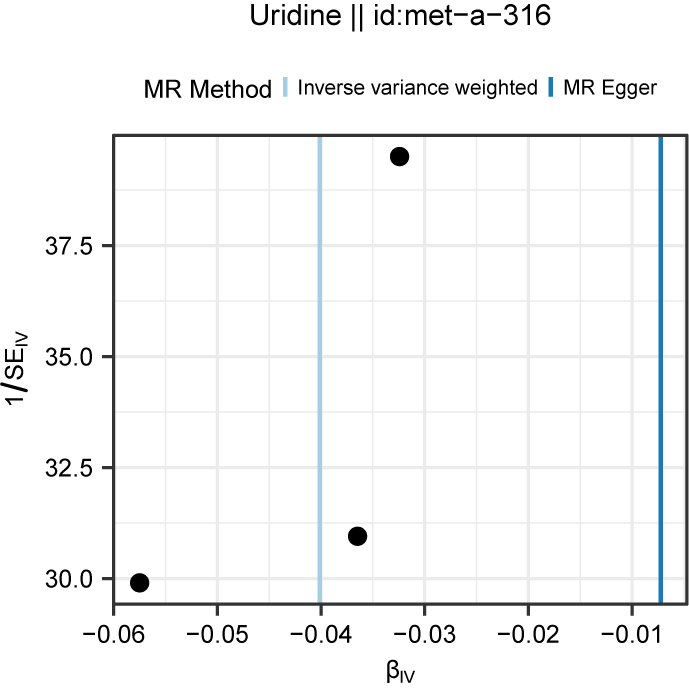

Supplement: Supplementary file 1 [file DataSheet1.zip › Supplementary Figures/Figure S4 Funnel plot of heterogeneity test for MR of uridine in osteoporosis..tif]

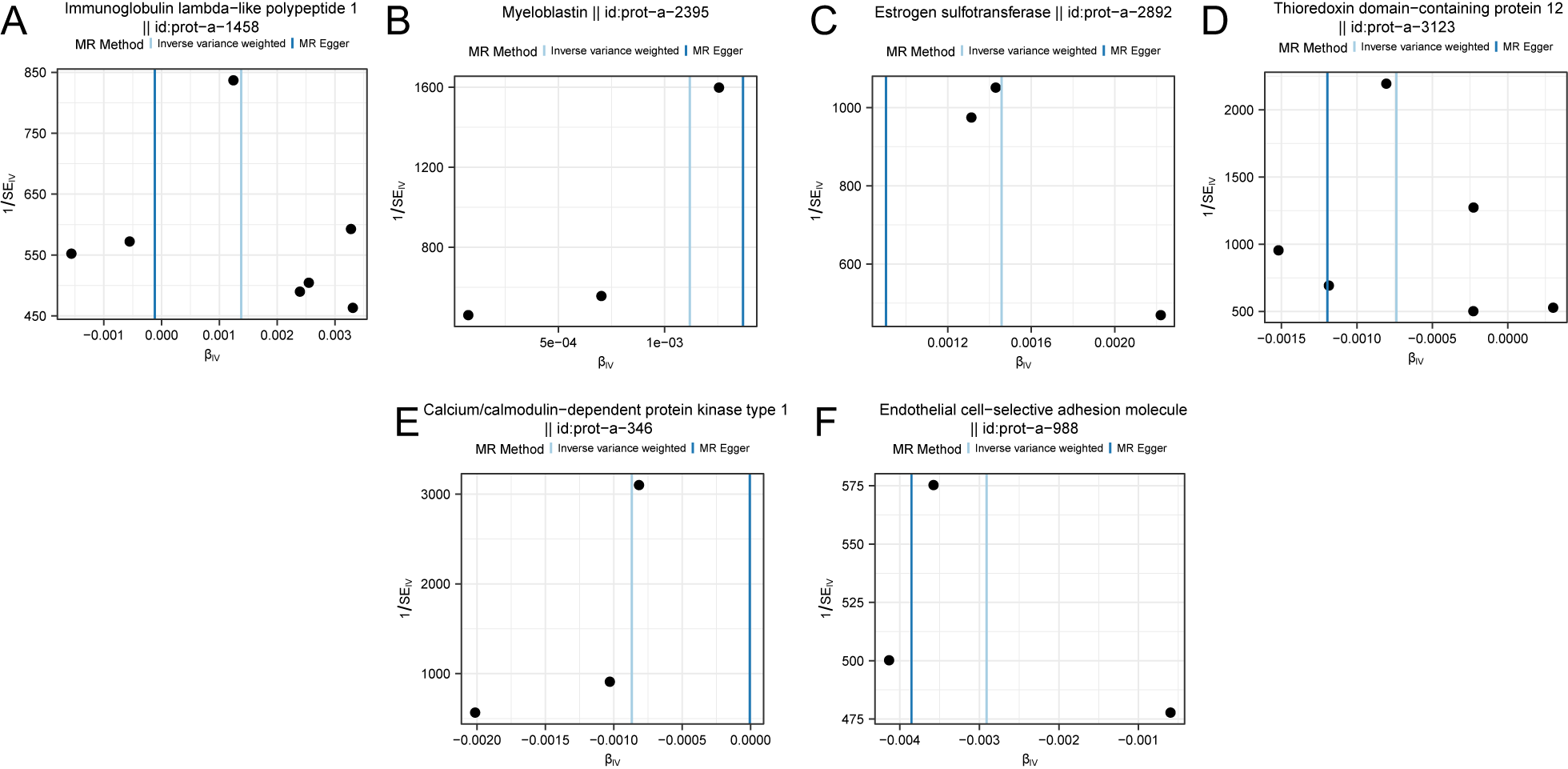

Supplement: Supplementary file 1 [file DataSheet1.zip › Supplementary Figures/Figure S3 Funnel plot of heterogeneity test for MR of plasma proteins in osteoporosis..tif]

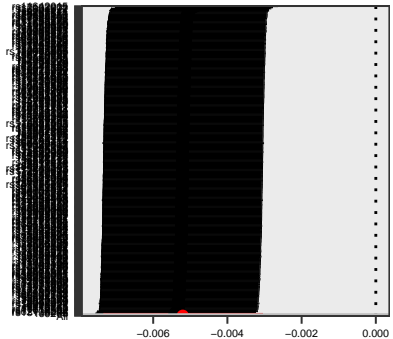

Supplement: Supplementary file 4 [file DataSheet4.zip › mr_leaveoneout_plot_ΦéÑΦâûμîçμáç/mr_leaveoneout_plot-ukb-a-24820-ukb-a-87.pdf]

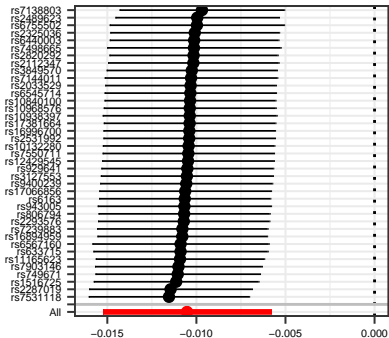

Supplement: Supplementary file 4 [file DataSheet4.zip › mr_leaveoneout_plot_ΦéÑΦâûμîçμáç/mr_leaveoneout_plot-ieu-a-616-ukb-a-87.pdf]

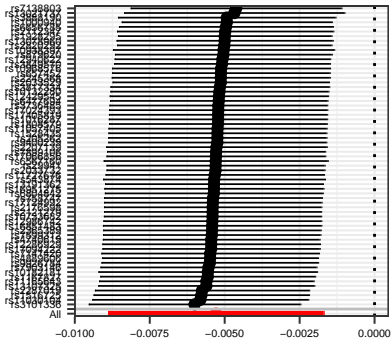

Supplement: Supplementary file 4 [file DataSheet4.zip › mr_leaveoneout_plot_ΦéÑΦâûμîçμáç/mr_leaveoneout_plot-ieu-a-83515-ukb-a-87.pdf]
