# Supplementary material for: Causal inference study of plasma proteins and blood metabolites mediating the effect of obesity-related indicators on osteoporosis
Source: Front Endocrinol (Lausanne). 2025 Feb 18;16:1435295. doi: 10.3389/fendo.2025.1435295 (PMC11876022; doi:10.3389/fendo.2025.1435295)
Supplement: Supplementary file 2 [file DataSheet2.zip › Supplementary Tables/Table S4 Instrumental variables of obesity-related indicators on osteoporosis.docx]

Table S4. **Instrumental variables screening of obesity-related indicators on osteoporosis and F test of instrumental variables**

| **Exposure** | **Number**  **of SNPs** | **Median of F** | **Minimum of F** | **Maximum**  **of F** |
| --- | --- | --- | --- | --- |
| **Body mass index \|\| id:ebi-a-GCST006368** | 141 | 40.87 | 29.78 | 311 |
| **Waist circumference \|\| id:ieu-a-103** | 2 | 68.2 | 41.95 | 94.44 |
| **Waist circumference \|\| id:ieu-a-105** | 4 | 36.52 | 33.71 | 39.74 |
| **Waist-to-hip ratio \|\| id:ieu-a-109** | 5 | 37.87 | 31.02 | 49.59 |
| **Waist-to-hip ratio \|\| id:ieu-a-111** | 7 | 45.25 | 30.33 | 63.06 |
| **Waist circumference \|\| id:ieu-a-61** | 39 | 38.15 | 29.34 | 144 |
| **Waist circumference \|\| id:ieu-a-63** | 16 | 40.83 | 29.75 | 103.88 |
| **Waist circumference \|\| id:ieu-a-65** | 13 | 50.17 | 31.07 | 268.4 |
| **Waist circumference \|\| id:ieu-a-69** | 21 | 47.1 | 30.36 | 130.61 |
| **Waist circumference \|\| id:ieu-a-71** | 25 | 44.44 | 29.96 | 86.22 |
| **Waist-to-hip ratio \|\| id:ieu-a-73** | 28 | 39.94 | 29.75 | 169.79 |
| **Waist-to-hip ratio \|\| id:ieu-a-75** | 22 | 40.69 | 30.36 | 108.88 |
| **Body mass index \|\| id:ieu-a-785** | 28 | 41.87 | 29.73 | 419.74 |
| **Waist-to-hip ratio \|\| id:ieu-a-81** | 32 | 43.55 | 29.16 | 153.76 |
| **Body mass index \|\| id:ieu-a-835** | 65 | 41.62 | 29.02 | 238.53 |
| **Body mass index \|\| id:ieu-a-94** | 7 | 33 | 31.82 | 176.39 |
| **Body mass index \|\| id:ieu-a-95** | 7 | 50.08 | 30.07 | 145.52 |
| **Body mass index \|\| id:ieu-a-974** | 35 | 43.89 | 29.64 | 393.87 |
| **body mass index \|\| id:ieu-b-40** | 446 | 51.43 | 28.62 | 1426.17 |
| **Body mass index (BMI) \|\| id:ukb-a-248** | 275 | 42.81 | 29.78 | 874.39 |
| **Leg fat percentage (right) \|\| id:ukb-a-274** | 222 | 42.41 | 29.73 | 424.74 |
| **Leg fat percentage (left) \|\| id:ukb-a-278** | 221 | 41.68 | 29.74 | 425.35 |
| **Arm fat percentage (right) \|\| id:ukb-a-282** | 213 | 41.65 | 29.75 | 546.49 |
| **Arm fat percentage (left) \|\| id:ukb-a-286** | 231 | 40.79 | 29.76 | 565.63 |
| **Waist circumference \|\| id:ukb-a-382** | 200 | 42.02 | 29.76 | 660.76 |
| **Arm fat percentage (right) \|\| id:ukb-b-12854** | 338 | 43.76 | 29.75 | 856.84 |
| **Body mass index (BMI) \|\| id:ukb-b-19953** | 379 | 47.45 | 29.76 | 1331.57 |
| **Arm fat percentage (left) \|\| id:ukb-b-20188** | 342 | 44.62 | 29.86 | 897.98 |
| **Body mass index (BMI) \|\| id:ukb-b-2303** | 374 | 47.38 | 29.76 | 1306.49 |
| **Body fat percentage \|\| id:ukb-b-8909** | 333 | 44.64 | 29.8 | 681.93 |
| **Waist circumference \|\| id:ukb-b-9405** | 320 | 42.84 | 29.85 | 997.38 |

SNPs：Single Nucleotide Polymorphisms；F：F statistics.
