# Supplementary material for: Causal inference study of plasma proteins and blood metabolites mediating the effect of obesity-related indicators on osteoporosis
Source: Front Endocrinol (Lausanne). 2025 Feb 18;16:1435295. doi: 10.3389/fendo.2025.1435295 (PMC11876022; doi:10.3389/fendo.2025.1435295)
Supplement: Supplementary file 2 [file DataSheet2.zip › Supplementary Tables/Table S14 Pleiotropy test of MR of obesity-related indicators.docx]

Table S14. **Pleiotropy test of MR analysis of obesity-related indicators for osteoporosis**

| **Exposure** | **MR-Egger intercept** | **Standard error** | **pvalue** |
| --- | --- | --- | --- |
| **Body mass index \|\| id：ebi-a-GCST006368** | -6.31E-05 | 9.23E-05 | 0.495641 |
| **Waist circumference \|\| id：ieu-a-105** | -0.0004 | 0.001017 | 0.73066 |
| **Waist-to-hip ratio \|\| id：ieu-a-109** | 0.00013 | 0.002444 | 0.960951 |
| **Waist-to-hip ratio \|\| id：ieu-a-111** | 0.000211 | 0.001051 | 0.848858 |
| **Waist circumference \|\| id：ieu-a-61** | -0.00014 | 0.000229 | 0.553508 |
| **Waist circumference \|\| id：ieu-a-63** | 6.24E-05 | 0.000386 | 0.873925 |
| **Waist circumference \|\| id：ieu-a-65** | 0.00059 | 0.000337 | 0.107572 |
| **Waist circumference \|\| id：ieu-a-69** | 0.000856 | 0.000434 | 0.063123 |
| **Waist circumference \|\| id：ieu-a-71** | -0.00021 | 0.000434 | 0.640459 |
| **Waist-to-hip ratio \|\| id：ieu-a-73** | 0.00027 | 0.00032 | 0.405727 |
| **Waist-to-hip ratio \|\| id：ieu-a-75** | 0.000662 | 0.000432 | 0.140852 |
| **Body mass index \|\| id：ieu-a-785** | 0.00027 | 0.00023 | 0.250834 |
| **Waist-to-hip ratio \|\| id：ieu-a-81** | 0.000437 | 0.000288 | 0.13998 |
| **Body mass index \|\| id：ieu-a-835** | -0.00011 | 0.000146 | 0.450246 |
| **Body mass index \|\| id：ieu-a-94** | 0.000395 | 0.000443 | 0.412968 |
| **Body mass index \|\| id：ieu-a-95** | 0.000518 | 0.000469 | 0.319961 |
| **Body mass index \|\| id：ieu-a-974** | 0.00014 | 0.000182 | 0.446842 |
| **body mass index \|\| id：ieu-b-40** | -1.97E-05 | 4.70E-05 | 0.675919 |
| **Body mass index （BMI） \|\| id：ukb-a-248** | 7.67E-05 | 6.48E-05 | 0.237666 |
| **Leg fat percentage （right） \|\| id：ukb-a-274** | 0.000164 | 9.79E-05 | 0.094408 |
| **Leg fat percentage （left） \|\| id：ukb-a-278** | 3.42E-05 | 8.82E-05 | 0.698262 |
| **Arm fat percentage （right） \|\| id：ukb-a-282** | 7.17E-05 | 8.36E-05 | 0.392369 |
| **Arm fat percentage （left） \|\| id：ukb-a-286** | 6.92E-05 | 8.18E-05 | 0.398372 |
| **Waist circumference \|\| id：ukb-a-382** | 0.000131 | 8.55E-05 | 0.127209 |
| **Arm fat percentage （right） \|\| id：ukb-b-12854** | 0.000112 | 6.31E-05 | 0.077973 |
| **Body mass index （BMI） \|\| id：ukb-b-19953** | 6.08E-05 | 5.55E-05 | 0.273926 |
| **Arm fat percentage （left） \|\| id：ukb-b-20188** | 0.000104 | 6.33E-05 | 0.100726 |
| **Body mass index （BMI） \|\| id：ukb-b-2303** | 7.71E-05 | 5.55E-05 | 0.165377 |
| **Body fat percentage \|\| id：ukb-b-8909** | 6.19E-05 | 7.42E-05 | 0.405197 |
| **Waist circumference \|\| id：ukb-b-9405** | 4.22E-05 | 6.53E-05 | 0.518592 |
