# Supplementary material for: Causal inference study of plasma proteins and blood metabolites mediating the effect of obesity-related indicators on osteoporosis
Source: Front Endocrinol (Lausanne). 2025 Feb 18;16:1435295. doi: 10.3389/fendo.2025.1435295 (PMC11876022; doi:10.3389/fendo.2025.1435295)
Supplement: Supplementary file 2 [file DataSheet2.zip › Supplementary Tables/Table S5 Instrumental variables of Plasma protein on osteoporosis.docx]

Table S5. **Instrumental variables screening of Plasma protein on osteoporosis and F test of instrumental variables**

| **Exposure** | **Number of SNPs** | **Median of F** | **Minimum of F** | **Maximum of F** |
| --- | --- | --- | --- | --- |
| **Ankyrin repeat domain-containing protein 46 \|\| id：prot-a-103** | 2 | 30.63 | 30.12 | 31.15 |
| **Apolipoprotein M \|\| id：prot-a-136** | 2 | 55.52 | 48.67 | 62.36 |
| 1. **type lectin domain family 12 member A \|\|**   **id：prot-a-570** | 2 | 4550.75 | 50.29 | 9051.21 |
| **Calcium/calmodulin-dependent protein kinase type 1 \|\| id：prot-a-346** | 3 | 122.38 | 40.21 | 2377.28 |
| **Carbonic anhydrase 9 \|\| id：prot-a-334** | 2 | 33.55 | 30.07 | 37.04 |
| **Chordin-like protein 2 \|\| id：prot-a-549** | 2 | 80.83 | 50.63 | 111.03 |
| **Ecto-ADP-ribosyltransferase 4 \|\| id：prot-a-176** | 2 | 596.27 | 30.34 | 1162.2 |
| **Endothelial cell-selective adhesion molecule \|\|**  **id：prot-a-988** | 3 | 37.13 | 30.79 | 51.55 |
| **Estrogen sulfotransferase \|\| id：prot-a-2892** | 3 | 148.88 | 33.02 | 164.57 |
| **Glutamate receptor ionotropic， delta-2 \|\|**  **id：prot-a-1276** | 2 | 74.44 | 31.63 | 117.25 |
| **Histone-lysine N-methyltransferase EHMT2 \|\| id：prot-a-914** | 2 | 46.3 | 39.7 | 52.89 |
| **Immunoglobulin lambda-like polypeptide 1 \|\|**  **id：prot-a-1458** | 7 | 46.78 | 32.66 | 109.8 |
| **Interleukin-17 receptor B \|\| id：prot-a-1487** | 2 | 201.01 | 56.94 | 345.07 |
| **Killer cell lectin-like receptor subfamily F member 1 \|\| id：prot-a-1673** | 2 | 39.13 | 31.5 | 46.75 |
| **Lactoperoxidase \|\| id：prot-a-1765** | 2 | 66.16 | 57.53 | 74.78 |
| **Myeloblastin \|\| id：prot-a-2395** | 3 | 44.95 | 35.97 | 435.33 |
| **NKG2-E type II integral membrane protein \|\|**  **id：prot-a-1671** | 2 | 148.83 | 32.5 | 265.17 |
| **Neural cell adhesion molecule 2 \|\|**  **id：prot-a-2008** | 2 | 58.37 | 54.95 | 61.8 |
| **Platelet-derived growth factor receptor alpha \|\| id：prot-a-2229** | 2 | 288.48 | 29.88 | 547.09 |
| **Potassium-transporting ATPase subunit beta \|\| id：prot-a-202** | 2 | 45.65 | 42.6 | 48.69 |
| **Serine/threonine-protein kinase pim-1 \|\|**  **id：prot-a-2274** | 2 | 94.18 | 31.21 | 157.15 |
| **Thioredoxin domain-containing protein 12 \|\|**  **id：prot-a-3123** | 6 | 111.46 | 44.95 | 951.38 |
| **Transcobalamin-1 \|\| id：prot-a-2938** | 2 | 110.11 | 90.51 | 129.71 |
| **Transforming growth factor-beta-induced protein ig-h3 \|\| id：prot-a-2966** | 2 | 189.52 | 30.85 | 348.18 |
| **Zinc finger protein 175 \|\| id：prot-a-3262** | 2 | 163.07 | 30.18 | 295.97 |

SNPs：Single Nucleotide Polymorphisms；F：F statistics.
