# Supplementary material for: Causal inference study of plasma proteins and blood metabolites mediating the effect of obesity-related indicators on osteoporosis
Source: Front Endocrinol (Lausanne). 2025 Feb 18;16:1435295. doi: 10.3389/fendo.2025.1435295 (PMC11876022; doi:10.3389/fendo.2025.1435295)
Supplement: Supplementary file 2 [file DataSheet2.zip › Supplementary Tables/Table S19 Heterogeneity test of MR of blood metabolites for osteoporosis .docx]

Table S19. **MR analysis of blood metabolites for osteoporosis heterogeneity test**

| **Exposure** | **Q** | **Q_df** | **Q_pval** | **I^2^（%）** |
| --- | --- | --- | --- | --- |
| **Uridine \|\| id：met-a-316** | 0.212651 | 1 | 0.644698 | 0 |
| **Uridine \|\| id：met-a-316** | 0.375105 | 2 | 0.828985 | 0 |
| **Alanine \|\| id：met-a-469** | 1.289553 | 1 | 0.25613 | 22.45 |
| **1-linoleoylglycerophosphoethanolamine* \|\| id：met-a-497** | 1.147427 | 1 | 0.284088 | 12.85 |
| **1-arachidonoylglycerophosphoinositol* \|\| id：met-a-634** | 0.065132 | 1 | 0.798561 | 0 |
| **Hexadecanedioate \|\| id：met-a-711** | 1.068939 | 1 | 0.301185 | 6.45 |
| **X-14626 \|\| id：met-a-729** | 0.5425 | 1 | 0.461398 | 0 |
| **4-androsten-3beta，17beta-diol disulfate 2* \|\| id：met-a-748** | 0.508347 | 1 | 0.475855 | 0 |

Q: Cochran Q test；Q_df: degrees of freedom of Q test; Q_pval: P valve of Q test
