# Supplementary material for: Causal inference study of plasma proteins and blood metabolites mediating the effect of obesity-related indicators on osteoporosis
Source: Front Endocrinol (Lausanne). 2025 Feb 18;16:1435295. doi: 10.3389/fendo.2025.1435295 (PMC11876022; doi:10.3389/fendo.2025.1435295)
Supplement: Supplementary file 2 [file DataSheet2.zip › Supplementary Tables/Table S6 Instrumental variables of blood metabolites on osteoporosis.docx]

Table S6. **Instrumental variables screening of blood metabolites on osteoporosis and F test of instrumental variables**

| **Exposure** | **Number of SNPs** | **Median of F** | **Minimum of F** | **Maximum**  **of F** |
| --- | --- | --- | --- | --- |
| 1. **arachidonoylglycerophosphoinositol* \|\|**   **id：met-a-634** | 2 | 105.57 | 76.21 | 134.93 |
| 1. **linoleoylglycerophosphoethanolamine* \|\|**   **id：met-a-497** | 2 | 97.72 | 37.43 | 158 |
| 1. **androsten-3beta，17beta-diol disulfate 2* \|\|**   **id：met-a-748** | 2 | 65.45 | 53.17 | 77.74 |
| **Alanine \|\| id：met-a-469** | 2 | 46.29 | 33.22 | 59.36 |
| **Hexadecanedioate \|\| id：met-a-711** | 2 | 154.42 | 79.82 | 229.02 |
| **Uridine \|\| id：met-a-316** | 3 | 31.22 | 30.57 | 65.77 |
| **X-14626 \|\| id：met-a-729** | 2 | 119.89 | 40.88 | 198.9 |

SNPs：Single Nucleotide Polymorphisms；F：F statistics.
