# Supplementary material for: Causal inference study of plasma proteins and blood metabolites mediating the effect of obesity-related indicators on osteoporosis
Source: Front Endocrinol (Lausanne). 2025 Feb 18;16:1435295. doi: 10.3389/fendo.2025.1435295 (PMC11876022; doi:10.3389/fendo.2025.1435295)
Supplement: Supplementary file 2 [file DataSheet2.zip › Supplementary Tables/Table S25 Sobel test of blood metabolites mediating the effect of obesity-related indicators on osteoporosis.docx]

Table S25. **Sobel test of blood metabolites mediating the effect of obesity-related indicators on osteoporosis**

| **Model** | **Z value** | **pvalue** |
| --- | --- | --- |
| **model21** | 0.654366 | 0.512876 |
| **model22** | -0.70251 | 0.482361 |
| **model24** | 0.245797 | 0.80584 |
| **model25** | 1.295818 | 0.195038 |
| **model26** | 1.075655 | 0.282081 |
| **model27** | -0.85796 | 0.390914 |
| **model28** | 0.566734 | 0.570895 |
| **model29** | -0.02808 | 0.977601 |
| **model30** | -0.08546 | 0.931895 |
| **model31** | -0.04886 | 0.961035 |
| **model32** | -0.00096 | 0.999235 |
| **model33** | 1.114633 | 0.265008 |
| **model34** | -0.05267 | 0.957992 |
