# Supplementary material for: Causal inference study of plasma proteins and blood metabolites mediating the effect of obesity-related indicators on osteoporosis
Source: Front Endocrinol (Lausanne). 2025 Feb 18;16:1435295. doi: 10.3389/fendo.2025.1435295 (PMC11876022; doi:10.3389/fendo.2025.1435295)
Supplement: Supplementary file 2 [file DataSheet2.zip › Supplementary Tables/Table S10 The Steiger directivity test of MR of obesity-related indicators on osteoporosis.docx]

Table S10. **The Steiger directivity test of Mendelian randomized analysis of obesity-related indicators on osteoporosis**

| **Exposure** | **SNP r^2^ exposure** | **SNP r^2^ outcome** | **Correct causal direction** | **Steiger pvalue** |
| --- | --- | --- | --- | --- |
| **Body mass index \|\|**  **id：ebi-a-GCST006368** | 0.020202 | 0.00047 | TRUE | 0 |
| **Waist circumference \|\| id：ieu-a-103** | 0.002856 | 2.20E-05 | TRUE | 2.80E-23 |
| **Waist-to-hip ratio \|\| id：ieu-a-109** | 0.004522 | 0.000104 | TRUE | 9.04E-29 |
| **Waist-to-hip ratio \|\| id：ieu-a-111** | 0.004733 | 7.11E-05 | TRUE | 5.43E-32 |
| **Waist circumference \|\| id：ieu-a-61** | 0.006375 | 0.000196 | TRUE | 4.19E-131 |
| **Waist circumference \|\| id：ieu-a-63** | 0.004824 | 0.00011 | TRUE | 6.27E-72 |
| **Waist circumference \|\| id：ieu-a-65** | 0.007967 | 7.51E-05 | TRUE | 4.84E-114 |
| **Waist circumference \|\| id：ieu-a-71** | 0.011086 | 0.000119 | TRUE | 2.55E-152 |
| **Waist-to-hip ratio \|\| id：ieu-a-73** | 0.00564 | 0.000101 | TRUE | 5.82E-121 |
| **Waist-to-hip ratio \|\| id：ieu-a-75** | 0.00939 | 0.000132 | TRUE | 2.81E-139 |
| **Body mass index \|\| id：ieu-a-785** | 0.010495 | 0.000166 | TRUE | 2.52E-184 |
| **Body mass index \|\| id：ieu-a-835** | 0.008967 | 0.000244 | TRUE | 2.28E-224 |
| **Body mass index \|\| id：ieu-a-94** | 0.006784 | 4.02E-05 | TRUE | 5.10E-65 |
| **Body mass index \|\| id：ieu-a-95** | 0.004442 | 4.12E-05 | TRUE | 2.97E-46 |
| **Body mass index \|\| id：ieu-a-974** | 0.009982 | 0.000133 | TRUE | 6.21E-193 |
| **body mass index \|\| id：ieu-b-40** | 0.038955 | 0.001695 | TRUE | 0 |
| **Body mass index （BMI） \|\|**  **id：ukb-a-248** | 0.022671 | 0.000607 | TRUE | 0 |
| **Body mass index （BMI） \|\|**  **id：ukb-b-19953** | 0.027173 | 0.001119 | TRUE | 0 |
| **Body mass index （BMI） \|\|**  **id：ukb-b-2303** | 0.026579 | 0.001067 | TRUE | 0 |
| **Waist circumference \|\|**  **id：ukb-b-9405** | 0.023459 | 0.001058 | TRUE | 0 |

SNP: single nucleotide polymorphism
