# Supplementary material for: Causal inference study of plasma proteins and blood metabolites mediating the effect of obesity-related indicators on osteoporosis
Source: Front Endocrinol (Lausanne). 2025 Feb 18;16:1435295. doi: 10.3389/fendo.2025.1435295 (PMC11876022; doi:10.3389/fendo.2025.1435295)
Supplement: Supplementary file 2 [file DataSheet2.zip › Supplementary Tables/Table S22 The Steiger directivity test of MR of obesity-related indicators and plasma proteins.docx]

Table S22. **The Steiger directivity test of MR of obesity-related indicators and plasma proteins**

| **Exposure** | **Outcome** | **SNP r^2^ exposure** | **SNP r^2^ outcome** | **Correct causal direction** | **Steiger pvalue** |
| --- | --- | --- | --- | --- | --- |
| **Waist circumference \|\| id：ieu-a-103** | Immunoglobulin lambda-like polypeptide 1 | 0.002856 | 0.002019 | TRUE | 0.636012 |
| **Waist-to-hip ratio \|\| id：ieu-a-109** | Calcium/calmodulin-dependent protein kinase type 1 | 0.004522 | 0.002317 | TRUE | 0.288586 |
| **Waist-to-hip ratio \|\| id：ieu-a-111** | Glutamate receptor ionotropic， delta-2 | 0.007484 | 0.001646 | TRUE | 0.010696 |
| **Waist-to-hip ratio \|\| id：ieu-a-111** | Thioredoxin domain-containing protein 12 | 0.007484 | 0.002739 | TRUE | 0.057393 |
| **Waist-to-hip ratio \|\| id：ieu-a-111** | Calcium/calmodulin-dependent protein kinase type 1 | 0.007484 | 0.004065 | TRUE | 0.205521 |
| **Waist circumference \|\| id：ieu-a-63** | Apolipoprotein M | 0.006196 | 0.005063 | TRUE | 0.666321 |
| **Waist circumference \|\| id：ieu-a-63** | Ecto-ADP-ribosyltransferase 4 | 0.006196 | 0.00493 | TRUE | 0.627853 |
| **Waist circumference \|\| id：ieu-a-63** | Neural cell adhesion molecule 2 | 0.006196 | 0.005858 | TRUE | 0.901233 |
| **Waist circumference \|\| id：ieu-a-63** | Histone-lysine N-methyltransferase EHMT2 | 0.006196 | 0.00524 | TRUE | 0.718163 |
| **Waist circumference \|\| id：ieu-a-69** | Neural cell adhesion molecule 2 | 0.008697 | 0.00814 | TRUE | 0.862393 |
| **Waist circumference \|\| id：ieu-a-69** | Histone-lysine N-methyltransferase EHMT2 | 0.008697 | 0.005779 | TRUE | 0.325084 |
| **Waist circumference \|\| id：ieu-a-71** | Platelet-derived growth factor receptor alpha | 0.011419 | 0.005968 | TRUE | 0.091573 |
| **Waist-to-hip ratio \|\| id：ieu-a-75** | Thioredoxin domain-containing protein 12 | 0.010273 | 0.008731 | TRUE | 0.650925 |
| **Waist-to-hip ratio \|\| id：ieu-a-75** | Zinc finger protein 175 | 0.010273 | 0.00745 | TRUE | 0.390264 |
| **Waist-to-hip ratio \|\| id：ieu-a-75** | Calcium/calmodulin-dependent protein kinase type 1 | 0.010273 | 0.009392 | TRUE | 0.799508 |
| **Waist-to-hip ratio \|\| id：ieu-a-75** | Histone-lysine N-methyltransferase EHMT2 | 0.010273 | 0.007292 | TRUE | 0.361897 |
| **Waist-to-hip ratio \|\| id：ieu-a-81** | Calcium/calmodulin-dependent protein kinase type 1 | 0.014988 | 0.011235 | TRUE | 0.345989 |
| **Waist-to-hip ratio \|\| id：ieu-a-81** | Histone-lysine N-methyltransferase EHMT2 | 0.014988 | 0.011361 | TRUE | 0.363635 |
| **Body mass index \|\| id：ieu-a-95** | Neural cell adhesion molecule 2 | 0.006489 | 0.004982 | TRUE | 0.573977 |
| **Body mass index \|\| id：ieu-a-974** | Neural cell adhesion molecule 2 | 0.01377 | 0.01048 | TRUE | 0.388726 |

SNP，single nucleotide polymorphism
