# Supplementary material for: Causal inference study of plasma proteins and blood metabolites mediating the effect of obesity-related indicators on osteoporosis
Source: Front Endocrinol (Lausanne). 2025 Feb 18;16:1435295. doi: 10.3389/fendo.2025.1435295 (PMC11876022; doi:10.3389/fendo.2025.1435295)
Supplement: Supplementary file 2 [file DataSheet2.zip › Supplementary Tables/Table S20 pleiotropy test of MR of blood metabolites for osteoporosis.docx]

Table S20. **Pleiotropy test of MR analysis of blood metabolites for osteoporosis**

| **Exposure** | **MR-Egger intercept** | **Standard error** | **pvalue** |
| --- | --- | --- | --- |
| **Uridine \|\| id：met-a-316** | -0.00037 | 0.000919764 | 0.75608626 |
