# Supplementary material for: Causal inference study of plasma proteins and blood metabolites mediating the effect of obesity-related indicators on osteoporosis
Source: Front Endocrinol (Lausanne). 2025 Feb 18;16:1435295. doi: 10.3389/fendo.2025.1435295 (PMC11876022; doi:10.3389/fendo.2025.1435295)
Supplement: Supplementary file 2 [file DataSheet2.zip › Supplementary Tables/Table S24 Sobel test of plasma protein mediating effects of obesity-related indicators on osteoporosis.docx]

Table S24. **Sobel test of plasma protein mediating effects of obesity-related indicators on osteoporosis**

| **Model** | **Z value** | **pvalue** |
| --- | --- | --- |
| **model2** | -0.74179 | 0.458214 |
| **model3** | -0.8682 | 0.385285 |
| **model4** | -1.1539 | 0.24854 |
| **model5** | -1.1271 | 0.259702 |
| **model6** | -0.15377 | 0.877793 |
| **model7** | -0.11916 | 0.905148 |
| **model8** | -1.14209 | 0.253415 |
| **model9** | 0.413514 | 0.67923 |
| **model10** | 0.166128 | 0.868056 |
| **model12** | 0.173172 | 0.862516 |
| **model13** | -1.57719 | 0.114751 |
| **model14** | -0.28586 | 0.774984 |
| **model15** | -1.13279 | 0.257301 |
| **model16** | -0.00989 | 0.992109 |
| **model17** | -0.6409 | 0.521587 |
| **model18** | -0.65644 | 0.511542 |
| **model19** | -1.00218 | 0.316255 |
| **model20** | -0.41735 | 0.676423 |
