# Supplementary material for: Causal inference study of plasma proteins and blood metabolites mediating the effect of obesity-related indicators on osteoporosis
Source: Front Endocrinol (Lausanne). 2025 Feb 18;16:1435295. doi: 10.3389/fendo.2025.1435295 (PMC11876022; doi:10.3389/fendo.2025.1435295)
Supplement: Supplementary file 2 [file DataSheet2.zip › Supplementary Tables/Table S16 Heterogeneity test of MR of plasma proteins for osteoporosis.docx]

Table S16. **MR analysis heterogeneity test of plasma proteins for osteoporosis**

| **Exposure** | **Q** | **Q_df** | **Q_pval** | **I^2^（%）** |
| --- | --- | --- | --- | --- |
| **Ankyrin repeat domain-containing protein 46 \|\|**  **id：prot-a-103** | 0.020021 | 1 | 0.887479 | 0 |
| **Glutamate receptor ionotropic， delta-2 \|\| id：prot-a-1276** | 0.251013 | 1 | 0.616362 | 0 |
| **Apolipoprotein M \|\| id：prot-a-136** | 0.000156 | 1 | 0.99004 | 0 |
| **Immunoglobulin lambda-like polypeptide 1 \|\| id：prot-a-1458** | 6.533776 | 6 | 0.36612 | 8.17 |
| **Interleukin-17 receptor B \|\| id：prot-a-1487** | 0.766982 | 1 | 0.381152 | 0 |
| **NKG2-E type II integral membrane protein \|\|**  **id：prot-a-1671** | 0.805256 | 1 | 0.369526 | 0 |
| **Killer cell lectin-like receptor subfamily F member 1 \|\|**  **id：prot-a-1673** | 1.798906 | 1 | 0.179845 | 44.41 |
| **Ecto-ADP-ribosyltransferase 4 \|\| id：prot-a-176** | 0.178406 | 1 | 0.672747 | 0 |
| **Lactoperoxidase \|\| id：prot-a-1765** | 0.45798 | 1 | 0.498569 | 0 |
| **Neural cell adhesion molecule 2 \|\| id：prot-a-2008** | 0.953639 | 1 | 0.328795 | 0 |
| **Potassium-transporting ATPase subunit beta \|\|**  **id：prot-a-202** | 0.365041 | 1 | 0.54572 | 0 |
| **Platelet-derived growth factor receptor alpha \|\|**  **id：prot-a-2229** | 0.761477 | 1 | 0.382867 | 0 |
| **Serine/threonine-protein kinase pim-1 \|\| id：prot-a-2274** | 0.145592 | 1 | 0.702784 | 0 |
| **Myeloblastin \|\| id：prot-a-2395** | 0.331576 | 2 | 0.847226 | 0 |
| **Estrogen sulfotransferase \|\| id：prot-a-2892** | 0.148662 | 2 | 0.928364 | 0 |
| **Transcobalamin-1 \|\| id：prot-a-2938** | 0.621055 | 1 | 0.430656 | 0 |
| **Transforming growth factor-beta-induced protein ig-h3 \|\| id：prot-a-2966** | 0.010804 | 1 | 0.917215 | 0 |
| **Thioredoxin domain-containing protein 12 \|\| id：prot-a-3123** | 1.464105 | 5 | 0.917175 | 0 |
| **Zinc finger protein 175 \|\| id：prot-a-3262** | 1.541974 | 1 | 0.214324 | 35.15 |
| **Carbonic anhydrase 9 \|\| id：prot-a-334** | 0.010839 | 1 | 0.917083 | 0 |
| **Calcium/calmodulin-dependent protein kinase type 1 \|\|**  **id：prot-a-346** | 0.467613 | 2 | 0.791515 | 0 |
| **Chordin-like protein 2 \|\| id：prot-a-549** | 1.505575 | 1 | 0.219816 | 33.58 |
| **C-type lectin domain family 12 member A \|\| id：prot-a-570** | 0.568809 | 1 | 0.450732 | 0 |
| **Histone-lysine N-methyltransferase EHMT2 \|\| id：prot-a-914** | 0.000762 | 1 | 0.977972 | 0 |
| **Endothelial cell-selective adhesion molecule \|\| id：prot-a-988** | 1.743606 | 2 | 0.418197 | 0 |
| **C-C motif chemokine ligand 4 \|\| id：prot-b-50** | 1.30296 | 2 | 0.521274 | 0 |

Q: Cochran Q test；Q_df: degrees of freedom of Q test; Q_pval: P valve of Q test
