# Supplementary material for: Causal inference study of plasma proteins and blood metabolites mediating the effect of obesity-related indicators on osteoporosis
Source: Front Endocrinol (Lausanne). 2025 Feb 18;16:1435295. doi: 10.3389/fendo.2025.1435295 (PMC11876022; doi:10.3389/fendo.2025.1435295)
Supplement: Supplementary file 2 [file DataSheet2.zip › Supplementary Tables/Table S17 Pleiotropy test of MR of plasma proteins for osteoporosis.docx]

Table S17. **Pleiotropy test of MR analysis of plasma proteins for osteoporosis**

| **Exposure** | **MR-Egger intercept** | **Standard error** | **pvalue** |
| --- | --- | --- | --- |
| **Immunoglobulin lambda-like polypeptide 1 \|\|**  **id：prot-a-1458** | 0.000454 | 0.000328636 | 0.225955957 |
| **Myeloblastin \|\| id：prot-a-2395** | -0.00012 | 0.000501306 | 0.854508111 |
| **Estrogen sulfotransferase \|\| id：prot-a-2892** | 0.000187 | 0.000492508 | 0.769434012 |
| **Thioredoxin domain-containing protein 12 \|\|**  **id：prot-a-3123** | 0.000447 | 0.000892209 | 0.64285583 |
| **Calcium/calmodulin-dependent protein kinase type 1 \|\| id：prot-a-346** | -0.00085 | 0.001711312 | 0.706750241 |
| **Endothelial cell-selective adhesion molecule \|\|**  **id：prot-a-988** | 0.000265 | 0.00095999 | 0.828766778 |
| **C-C motif chemokine ligand 4 \|\| id：prot-b-50** | -1.09E-05 | 0.003274232 | 0.99788127 |
