# Supplementary material for: Causal inference study of plasma proteins and blood metabolites mediating the effect of obesity-related indicators on osteoporosis
Source: Front Endocrinol (Lausanne). 2025 Feb 18;16:1435295. doi: 10.3389/fendo.2025.1435295 (PMC11876022; doi:10.3389/fendo.2025.1435295)
Supplement: Supplementary file 2 [file DataSheet2.zip › Supplementary Tables/Table S11 Steiger directivity test of MR of plasma proteins for osteoporosis.docx]

Table S11. **Mendelian randomization analysis of plasma proteins for osteoporosis Steiger directivity test**

| **Exposure** | **SNP r^2^ exposure** | **SNP r^2^ outcome** | **Correct causal direction** | **Steiger pvalue** |
| --- | --- | --- | --- | --- |
| **Ankyrin repeat domain-containing protein 46 \|\| id：prot-a-103** | 0.018498 | 1.48E-05 | TRUE | 2.92E-14 |
| **Glutamate receptor ionotropic， delta-2 \|\| id：prot-a-1276** | 0.044511 | 1.42E-05 | TRUE | 2.60E-33 |
| **Apolipoprotein M \|\| id：prot-a-136** | 0.033372 | 1.16E-05 | TRUE | 3.60E-25 |
| **Immunoglobulin lambda-like polypeptide 1 \|\| id：prot-a-1458** | 0.108308 | 3.29E-05 | TRUE | 3.22E-82 |
| **Interleukin-17 receptor B \|\| id：prot-a-1487** | 0.116647 | 2.18E-05 | TRUE | 1.38E-89 |
| **NKG2-E type II integral membrane protein \|\| id：prot-a-1671** | 0.087118 | 1.53E-05 | TRUE | 5.11E-66 |
| **Killer cell lectin-like receptor subfamily F member 1 \|\| id：prot-a-1673** | 0.0236 | 2.59E-05 | TRUE | 1.14E-17 |
| **Ecto-ADP-ribosyltransferase 4 \|\| id：prot-a-176** | 0.251123 | 1.52E-05 | TRUE | 1.92E-214 |
| **Lactoperoxidase \|\| id：prot-a-1765** | 0.039764 | 3.17E-05 | TRUE | 2.94E-29 |
| **Neural cell adhesion molecule 2 \|\| id：prot-a-2008** | 0.035072 | 1.69E-05 | TRUE | 3.13E-26 |
| **Potassium-transporting ATPase subunit beta \|\| id：prot-a-202** | 0.027463 | 1.51E-05 | TRUE | 9.89E-21 |
| **Platelet-derived growth factor receptor alpha \|\| id：prot-a-2229** | 0.162047 | 1.70E-05 | TRUE | 7.62E-129 |
| **Serine/threonine-protein kinase pim-1 \|\| id：prot-a-2274** | 0.05601 | 4.93E-05 | TRUE | 7.35E-41 |
| **Myeloblastin \|\| id：prot-a-2395** | 0.147934 | 1.24E-05 | TRUE | 9.03E-117 |
| **Estrogen sulfotransferase \|\| id：prot-a-2892** | 0.102931 | 1.48E-05 | TRUE | 9.73E-79 |
| **Transcobalamin-1 \|\| id：prot-a-2938** | 0.065668 | 1.78E-05 | TRUE | 3.68E-49 |
| **Transforming growth factor-beta-induced protein ig-h3 \|\| id：prot-a-2966** | 0.10991 | 1.19E-05 | TRUE | 1.25E-84 |
| **Thioredoxin domain-containing protein 12 \|\| id：prot-a-3123** | 0.41626 | 1.79E-05 | TRUE | 0 |
| **Zinc finger protein 175 \|\| id：prot-a-3262** | 0.094928 | 4.32E-05 | TRUE | 4.64E-71 |
| **Carbonic anhydrase 9 \|\| id：prot-a-334** | 0.020259 | 1.21E-05 | TRUE | 1.34E-15 |
| **Calcium/calmodulin-dependent protein kinase type 1 \|\| id：prot-a-346** | 0.29057 | 2.54E-05 | TRUE | 8.85E-256 |
| **Chordin-like protein 2 \|\| id：prot-a-549** | 0.04835 | 2.82E-05 | TRUE | 1.07E-35 |
| **C-type lectin domain family 12 member A \|\| id：prot-a-570** | 0.257116 | 2.99E-05 | TRUE | 1.90E-219 |
| **Histone-lysine N-methyltransferase EHMT2 \|\| id：prot-a-914** | 0.027896 | 1.15E-05 | TRUE | 3.68E-21 |
| **Endothelial cell-selective adhesion molecule \|\| id：prot-a-988** | 0.036013 | 2.55E-05 | TRUE | 1.14E-26 |

SNP，single nucleotide polymorphism
