# Supplementary material for: Causal inference study of plasma proteins and blood metabolites mediating the effect of obesity-related indicators on osteoporosis
Source: Front Endocrinol (Lausanne). 2025 Feb 18;16:1435295. doi: 10.3389/fendo.2025.1435295 (PMC11876022; doi:10.3389/fendo.2025.1435295)
Supplement: Supplementary file 2 [file DataSheet2.zip › Supplementary Tables/Table S23 The Steiger directivity test of MR of obesity-related indicators and blood metabolites.docx]

Table S23. **The Steiger directivity test of MR of obesity-related indicators and blood metabolites**

| **Exposure** | **Outcome** | **SNP r^2^ exposure** | **SNP r^2^ outcome** | **Correct causal direction** | **Steiger pvalue** |
| --- | --- | --- | --- | --- | --- |
| **Body mass index \|\| id：ebi-a-GCST006368** | 1-linoleoylglycerophosphoethanolamine* | 0.024288 | 0.01914 | TRUE | 0.134525 |
| **Waist circumference \|\| id：ieu-a-103** | 1-arachidonoylglycerophosphoinositol* | 0.002856 | 0.000831 | TRUE | 0.049209 |
| **Waist circumference \|\| id：ieu-a-105** | Alanine | 0.003054 | 0.001388 | TRUE | 0.150149 |
| **Waist circumference \|\| id：ieu-a-63** | 1-linoleoylglycerophosphoethanolamine* | 0.006196 | 0.002426 | TRUE | 0.015847 |
| **Waist circumference \|\| id：ieu-a-65** | Uridine | 0.007967 | 0.001547 | TRUE | 5.38E-05 |
| **Body mass index \|\| id：ieu-a-835** | 1-linoleoylglycerophosphoethanolamine* | 0.012064 | 0.007928 | TRUE | 0.07814 |
| **Body mass index \|\| id：ieu-a-94** | 1-arachidonoylglycerophosphoinositol* | 0.006784 | 0.001831 | TRUE | 0.001322 |
| **Body mass index \|\| id：ieu-a-95** | 1-linoleoylglycerophosphoethanolamine* | 0.006489 | 0.001425 | TRUE | 0.000889 |
| **Body mass index \|\| id：ieu-a-974** | 1-arachidonoylglycerophosphoinositol* | 0.01377 | 0.00502 | TRUE | 9.81E-05 |
| **Leg fat percentage （left） \|\| id：ukb-a-278** | 1-linoleoylglycerophosphoethanolamine* | 0.026633 | 0.024891 | TRUE | 0.643333 |
| **Arm fat percentage （right） \|\| id：ukb-a-282** | 1-linoleoylglycerophosphoethanolamine* | 0.027568 | 0.027172 | TRUE | 0.919824 |
| **Arm fat percentage （left） \|\| id：ukb-a-286** | 1-linoleoylglycerophosphoethanolamine* | 0.030072 | 0.029519 | TRUE | 0.892746 |
| **Waist circumference \|\| id：ukb-a-382** | 1-linoleoylglycerophosphoethanolamine* | 0.025983 | 0.02541 | TRUE | 0.878939 |
| **Body fat percentage \|\| id：ukb-b-8909** | Uridine | 0.035701 | 0.03079 | TRUE | 0.242768 |

SNP，single nucleotide polymorphism
