# Supplementary material for: Causal inference study of plasma proteins and blood metabolites mediating the effect of obesity-related indicators on osteoporosis
Source: Front Endocrinol (Lausanne). 2025 Feb 18;16:1435295. doi: 10.3389/fendo.2025.1435295 (PMC11876022; doi:10.3389/fendo.2025.1435295)
Supplement: Supplementary file 2 [file DataSheet2.zip › Supplementary Tables/Table S13 Heterogeneity test of MR of obesity-related indicators for osteoporosis.docx]

Table S13. **MR analysis heterogeneity test of obesity-related indicators for osteoporosis**

| **Exposure** | **Q** | **Q_df** | **Q_pval** | **I^2^（%）** |
| --- | --- | --- | --- | --- |
| **Body mass index \|\| id：ebi-a-GCST006368** | 164.1292 | 140 | 0.079875 | 14.7 |
| **Waist circumference \|\| id：ieu-a-103** | 0.758966 | 1 | 0.383652 | 0 |
| **Waist circumference \|\| id：ieu-a-105** | 4.320423 | 3 | 0.228878 | 30.56 |
| **Waist-to-hip ratio \|\| id：ieu-a-109** | 1.338095 | 4 | 0.85488 | 0 |
| **Waist-to-hip ratio \|\| id：ieu-a-111** | 9.205695 | 6 | 0.162336 | 34.82 |
| **Waist circumference \|\| id：ieu-a-61** | 54.35881 | 38 | 0.041488 | 30.09 |
| **Waist circumference \|\| id：ieu-a-63** | 23.6919 | 15 | 0.070514 | 36.69 |
| **Waist circumference \|\| id：ieu-a-65** | 15.67116 | 12 | 0.206766 | 23.43 |
| **Waist circumference \|\| id：ieu-a-69** | 41.22443 | 20 | 0.003486 | 51.49 |
| **Waist circumference \|\| id：ieu-a-71** | 33.52136 | 24 | 0.093542 | 28.4 |
| **Waist-to-hip ratio \|\| id：ieu-a-73** | 31.96644 | 27 | 0.23333 | 15.54 |
| **Waist-to-hip ratio \|\| id：ieu-a-75** | 31.64875 | 21 | 0.063518 | 33.65 |
| **Body mass index \|\| id：ieu-a-785** | 48.19913 | 27 | 0.007294 | 43.98 |
| **Waist-to-hip ratio \|\| id：ieu-a-81** | 49.63311 | 31 | 0.01823 | 37.54 |
| **Body mass index \|\| id：ieu-a-835** | 87.80287 | 64 | 0.025855 | 27.11 |
| **Body mass index \|\| id：ieu-a-94** | 5.661531 | 6 | 0.462152 | 0 |
| **Body mass index \|\| id：ieu-a-95** | 3.863846 | 6 | 0.695096 | 0 |
| **Body mass index \|\| id：ieu-a-974** | 46.74705 | 34 | 0.071464 | 27.27 |
| **body mass index \|\| id：ieu-b-40** | 567.9076 | 445 | 6.84E-05 | 21.64 |
| **Body mass index （BMI） \|\| id：ukb-a-248** | 333.0109 | 274 | 0.008478 | 17.72 |
| **Leg fat percentage （right） \|\| id：ukb-a-274** | 297.9163 | 221 | 0.000427 | 25.82 |
| **Leg fat percentage （left） \|\| id：ukb-a-278** | 252.8908 | 220 | 0.063324 | 13.01 |
| **Arm fat percentage （right） \|\| id：ukb-a-282** | 263.0714 | 212 | 0.009723 | 19.41 |
| **Arm fat percentage （left） \|\| id：ukb-a-286** | 302.5054 | 230 | 0.000937 | 23.97 |
| **Waist circumference \|\| id：ukb-a-382** | 275.0541 | 199 | 0.000286 | 27.65 |
| **Arm fat percentage （right） \|\| id：ukb-b-12854** | 445.8741 | 337 | 6.23E-05 | 24.42 |
| **Body mass index （BMI） \|\| id：ukb-b-19953** | 524.6783 | 378 | 8.27E-07 | 27.96 |
| **Arm fat percentage （left） \|\| id：ukb-b-20188** | 460.1476 | 341 | 1.71E-05 | 25.89 |
| **Body mass index （BMI） \|\| id：ukb-b-2303** | 514.1582 | 373 | 1.62E-06 | 27.45 |
| **Body fat percentage \|\| id：ukb-b-8909** | 503.4387 | 332 | 3.55E-09 | 34.05 |
| **Waist circumference \|\| id：ukb-b-9405** | 480.8098 | 319 | 1.16E-08 | 33.65 |

Q: Cochran Q test；Q_df: degrees of freedom of Q test; Q_pval: P valve of Q test
