# Supplementary material for: Causal inference study of plasma proteins and blood metabolites mediating the effect of obesity-related indicators on osteoporosis
Source: Front Endocrinol (Lausanne). 2025 Feb 18;16:1435295. doi: 10.3389/fendo.2025.1435295 (PMC11876022; doi:10.3389/fendo.2025.1435295)
Supplement: Supplementary file 2 [file DataSheet2.zip › Supplementary Tables/Table S12 Steiger directionality test of MR of Blood metabolism on osteoporosis.docx]

Table S12. **Blood metabolism on osteoporosis MR analysis Steiger directionality test**

| **Exposure** | **SNP r^2^ exposure** | **SNP r^2^ outcome** | **Correct causal direction** | **Steiger pvalue** |
| --- | --- | --- | --- | --- |
| **Uridine \|\| id：met-a-316** | 0.017371 | 1.74E-05 | TRUE | 1.28E-27 |
| **Alanine \|\| id：met-a-469** | 0.012017 | 4.31E-05 | TRUE | 1.74E-18 |
| **1-linoleoylglycerophosphoethanolamine* \|\| id：met-a-497** | 0.026248 | 2.14E-05 | TRUE | 1.91E-41 |
| **1-arachidonoylglycerophosphoinositol* \|\| id：met-a-634** | 0.028175 | 2.33E-05 | TRUE | 2.69E-44 |
| **Hexadecanedioate \|\| id：met-a-711** | 0.047958 | 1.64E-05 | TRUE | 1.20E-67 |
| **X-14626 \|\| id：met-a-729** | 0.036646 | 1.57E-05 | TRUE | 1.26E-51 |
| **4-androsten-3beta，17beta-diol disulfate 2* \|\| id：met-a-748** | 0.017796 | 2.01E-05 | TRUE | 4.40E-28 |

SNP，single nucleotide polymorphism
