# Supplementary material for: Causal inference study of plasma proteins and blood metabolites mediating the effect of obesity-related indicators on osteoporosis
Source: Front Endocrinol (Lausanne). 2025 Feb 18;16:1435295. doi: 10.3389/fendo.2025.1435295 (PMC11876022; doi:10.3389/fendo.2025.1435295)
Supplement: Supplementary file 3 [file DataSheet3.zip › mr_leaveoneout_plot_ΦíÇμ╢▓Σ╗úΦ░óτë⌐/mr_leaveoneout_plot-met-a-316-ukb-a-871.pdf]

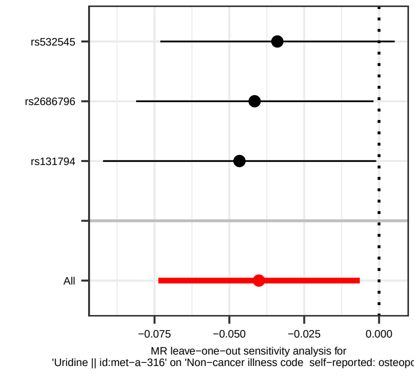
rs532545
rs2686796
rs131794
All
−0.075
−0.050
−0.025
0.000
MR leave−one−out sensitivity analysis for
'Uridine || id:met−a−316' on 'Non−cancer illness code  self−reported: osteopo
