# Supplementary material for: Causal inference study of plasma proteins and blood metabolites mediating the effect of obesity-related indicators on osteoporosis
Source: Front Endocrinol (Lausanne). 2025 Feb 18;16:1435295. doi: 10.3389/fendo.2025.1435295 (PMC11876022; doi:10.3389/fendo.2025.1435295)
Supplement: Supplementary file 4 [file DataSheet4.zip › mr_leaveoneout_plot_ΦéÑΦâûμîçμáç/mr_leaveoneout_plot-ukb-b-2018828-ukb-a-87.pdf]

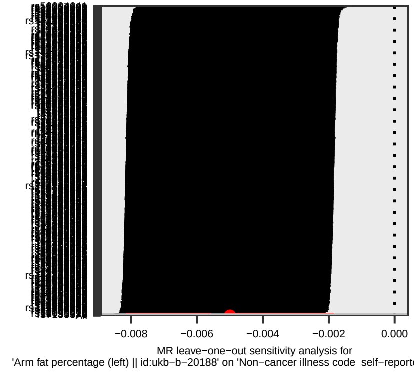

−0.008
−0.006
−0.004
−0.002
0.000
MR leave−one−out sensitivity analysis for
'Arm fat percentage (left) || id:ukb−b−20188' on 'Non−cancer illness code self−reporte
