# Supplementary material for: Causal inference study of plasma proteins and blood metabolites mediating the effect of obesity-related indicators on osteoporosis
Source: Front Endocrinol (Lausanne). 2025 Feb 18;16:1435295. doi: 10.3389/fendo.2025.1435295 (PMC11876022; doi:10.3389/fendo.2025.1435295)
Supplement: Supplementary file 4 [file DataSheet4.zip › mr_leaveoneout_plot_ΦéÑΦâûμîçμáç/mr_leaveoneout_plot-ieu-a-97418-ukb-a-87.pdf]

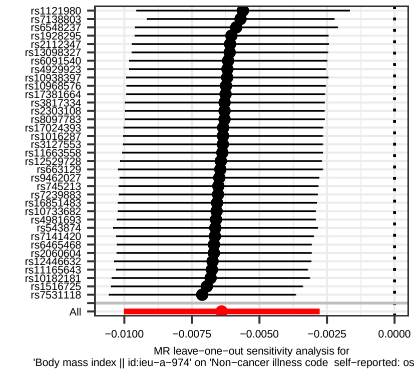

rs1121980
rs7138803
rs6548237
rs1928295
rs2112347
rs13098327
rs6091540
rs4929923
rs10938397
rs10968576
rs17381664
rs3817334
rs2303108
rs8097783
rs17024393
rs1016287
rs3127553
rs11663558
rs12529728
rs663129
rs9462027
rs745213
rs7239883
rs16851483
rs10733682
rs4981693
rs543874
rs7141420
rs6465468
rs2060604
rs12446632
rs11165643
rs10182181
rs1516725
rs7531118
All
−0.0100
−0.0075
−0.0050
−0.0025
0.0000
MR leave−one−out sensitivity analysis for
'Body mass index || id:ieu−a−974' on 'Non−cancer illness code self−reported: os
