# Supplementary material for: Causal inference study of plasma proteins and blood metabolites mediating the effect of obesity-related indicators on osteoporosis
Source: Front Endocrinol (Lausanne). 2025 Feb 18;16:1435295. doi: 10.3389/fendo.2025.1435295 (PMC11876022; doi:10.3389/fendo.2025.1435295)
Supplement: Supplementary file 4 [file DataSheet4.zip › mr_leaveoneout_plot_ΦéÑΦâûμîçμáç/mr_leaveoneout_plot-ieu-a-8114-ukb-a-87.pdf]

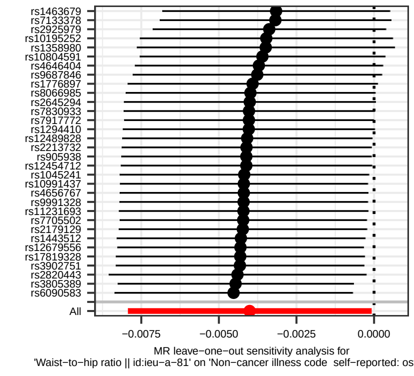
rs1463679
rs7133378
rs2925979
rs10195252
rs1358980
rs10804591
rs4646404
rs9687846
rs1776897
rs8066985
rs2645294
rs7830933
rs7917772
rs1294410
rs12489828
rs2213732
rs905938
rs12454712
rs1045241
rs10991437
rs4656767
rs9991328
rs11231693
rs7705502
rs2179129
rs1443512
rs12679556
rs17819328
rs3902751
rs2820443
rs3805389
rs6090583
All
−0.0075
−0.0050
−0.0025
0.0000
MR leave−one−out sensitivity analysis for
'Waist−to−hip ratio || id:ieu−a−81' on 'Non−cancer illness code self−reported: os
