# Supplementary material for: Causal inference study of plasma proteins and blood metabolites mediating the effect of obesity-related indicators on osteoporosis
Source: Front Endocrinol (Lausanne). 2025 Feb 18;16:1435295. doi: 10.3389/fendo.2025.1435295 (PMC11876022; doi:10.3389/fendo.2025.1435295)
Supplement: Supplementary file 4 [file DataSheet4.zip › mr_leaveoneout_plot_ΦéÑΦâûμîçμáç/mr_leaveoneout_plot-ieu-a-78513-ukb-a-87.pdf]

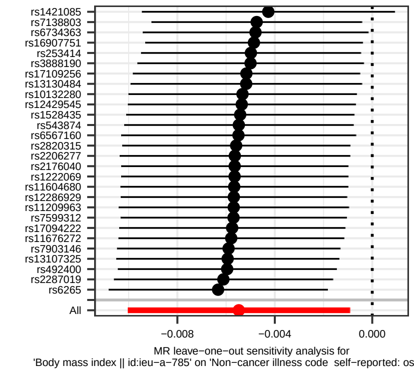

rs1421085
rs7138803
rs6734363
rs16907751
rs253414
rs3888190
rs17109256
rs13130484
rs10132280
rs12429545
rs1528435
rs543874
rs6567160
rs2820315
rs2206277
rs2176040
rs1222069
rs11604680
rs12286929
rs11209963
rs7599312
rs17094222
rs11676272
rs7903146
rs13107325
rs492400
rs2287019
rs6265
All
−0.008
−0.004
0.000
MR leave−one−out sensitivity analysis for
'Body mass index || id:ieu−a−785' on 'Non−cancer illness code self−reported: os
