# Supplementary material for: Causal inference study of plasma proteins and blood metabolites mediating the effect of obesity-related indicators on osteoporosis
Source: Front Endocrinol (Lausanne). 2025 Feb 18;16:1435295. doi: 10.3389/fendo.2025.1435295 (PMC11876022; doi:10.3389/fendo.2025.1435295)
Supplement: Supplementary file 4 [file DataSheet4.zip › mr_leaveoneout_plot_ΦéÑΦâûμîçμáç/mr_leaveoneout_plot-ieu-a-637-ukb-a-87.pdf]

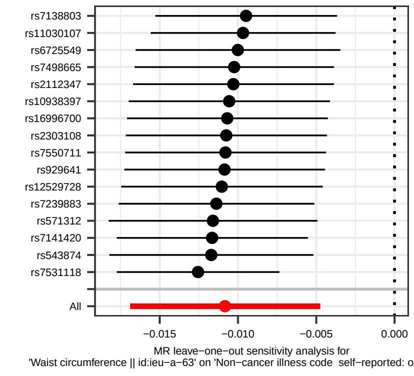
rs7138803
rs11030107
rs6725549
rs7498665
rs2112347
rs10938397
rs16996700
rs2303108
rs7550711
rs929641
rs12529728
rs7239883
rs571312
rs7141420
rs543874
rs7531118
All
−0.015
−0.010
−0.005
0.000
MR leave−one−out sensitivity analysis for
'Waist circumference || id:ieu−a−63' on 'Non−cancer illness code self−reported: o
