# Supplementary material for: Causal inference study of plasma proteins and blood metabolites mediating the effect of obesity-related indicators on osteoporosis
Source: Front Endocrinol (Lausanne). 2025 Feb 18;16:1435295. doi: 10.3389/fendo.2025.1435295 (PMC11876022; doi:10.3389/fendo.2025.1435295)
Supplement: Supplementary file 4 [file DataSheet4.zip › mr_leaveoneout_plot_ΦéÑΦâûμîçμáç/mr_leaveoneout_plot-ieu-a-7110-ukb-a-87.pdf]

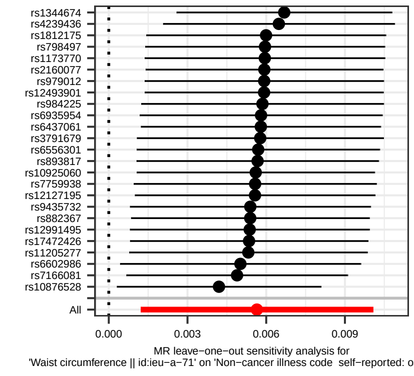
rs1344674
rs4239436
rs1812175
rs798497
rs1173770
rs2160077
rs979012
rs12493901
rs984225
rs6935954
rs6437061
rs3791679
rs6556301
rs893817
rs10925060
rs7759938
rs12127195
rs9435732
rs882367
rs12991495
rs17472426
rs11205277
rs6602986
rs7166081
rs10876528
All
0.000
0.003
0.006
0.009
MR leave−one−out sensitivity analysis for
'Waist circumference || id:ieu−a−71' on 'Non−cancer illness code self−reported: o
