# Supplementary material for: Causal inference study of plasma proteins and blood metabolites mediating the effect of obesity-related indicators on osteoporosis
Source: Front Endocrinol (Lausanne). 2025 Feb 18;16:1435295. doi: 10.3389/fendo.2025.1435295 (PMC11876022; doi:10.3389/fendo.2025.1435295)
Supplement: Supplementary file 4 [file DataSheet4.zip › mr_leaveoneout_plot_ΦéÑΦâûμîçμáç/mr_leaveoneout_plot-ieu-a-699-ukb-a-87.pdf]

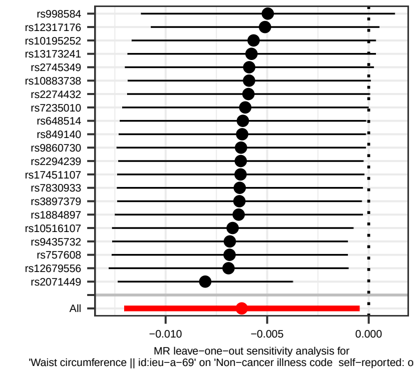
rs998584
rs12317176
rs10195252
rs13173241
rs2745349
rs10883738
rs2274432
rs7235010
rs648514
rs849140
rs9860730
rs2294239
rs17451107
rs7830933
rs3897379
rs1884897
rs10516107
rs9435732
rs757608
rs12679556
rs2071449
All
−0.010
−0.005
0.000
MR leave−one−out sensitivity analysis for
'Waist circumference || id:ieu−a−69' on 'Non−cancer illness code self−reported: o
