# Supplementary material for: Causal inference study of plasma proteins and blood metabolites mediating the effect of obesity-related indicators on osteoporosis
Source: Front Endocrinol (Lausanne). 2025 Feb 18;16:1435295. doi: 10.3389/fendo.2025.1435295 (PMC11876022; doi:10.3389/fendo.2025.1435295)
Supplement: Supplementary file 4 [file DataSheet4.zip › mr_leaveoneout_plot_ΦéÑΦâûμîçμáç/mr_leaveoneout_plot-ieu-a-9517-ukb-a-87.pdf]

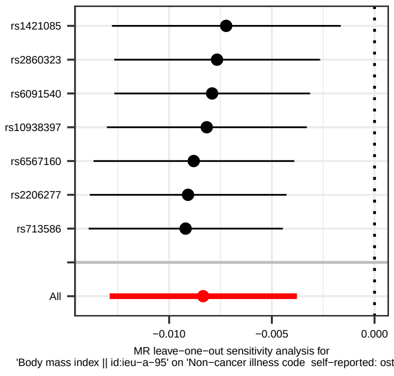
rs1421085
rs2860323
rs6091540
rs10938397
rs6567160
rs2206277
rs713586
All
−0.010
−0.005
0.000
MR leave−one−out sensitivity analysis for
'Body mass index || id:ieu−a−95' on 'Non−cancer illness code self−reported: ost
