# Supplementary material for: Causal inference study of plasma proteins and blood metabolites mediating the effect of obesity-related indicators on osteoporosis
Source: Front Endocrinol (Lausanne). 2025 Feb 18;16:1435295. doi: 10.3389/fendo.2025.1435295 (PMC11876022; doi:10.3389/fendo.2025.1435295)
Supplement: Supplementary file 4 [file DataSheet4.zip › mr_leaveoneout_plot_ΦéÑΦâûμîçμáç/mr_leaveoneout_plot-ukb-b-940531-ukb-a-87.pdf]

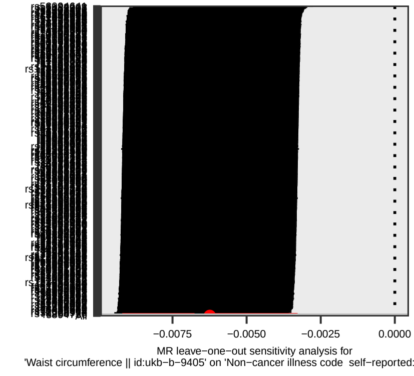
−0.0075
−0.0050
−0.0025
0.0000
MR leave−one−out sensitivity analysis for
'Waist circumference || id:ukb−b−9405' on 'Non−cancer illness code self−reported:
