# Supplementary material for: Causal inference study of plasma proteins and blood metabolites mediating the effect of obesity-related indicators on osteoporosis
Source: Front Endocrinol (Lausanne). 2025 Feb 18;16:1435295. doi: 10.3389/fendo.2025.1435295 (PMC11876022; doi:10.3389/fendo.2025.1435295)
Supplement: Supplementary file 4 [file DataSheet4.zip › mr_leaveoneout_plot_ΦéÑΦâûμîçμáç/mr_leaveoneout_plot-ieu-a-9416-ukb-a-87.pdf]

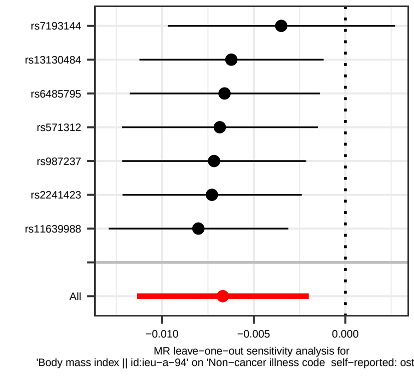
rs7193144
rs13130484
rs6485795
rs571312
rs987237
rs2241423
rs11639988
All
−0.010
−0.005
0.000
MR leave−one−out sensitivity analysis for
'Body mass index || id:ieu−a−94' on 'Non-cancer illness code self−reported: ost
