# Supplementary material for: Causal inference study of plasma proteins and blood metabolites mediating the effect of obesity-related indicators on osteoporosis
Source: Front Endocrinol (Lausanne). 2025 Feb 18;16:1435295. doi: 10.3389/fendo.2025.1435295 (PMC11876022; doi:10.3389/fendo.2025.1435295)
Supplement: Supplementary file 4 [file DataSheet4.zip › mr_leaveoneout_plot_ΦéÑΦâûμîçμáç/mr_leaveoneout_plot-ieu-a-7311-ukb-a-87.pdf]

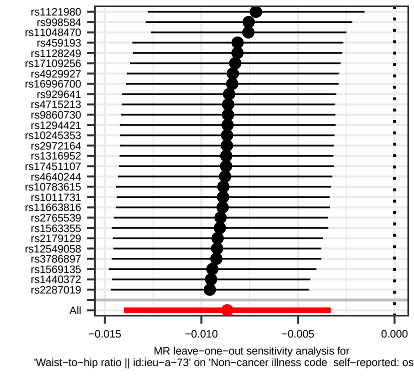

rs1121980
rs998584
rs11048470
rs459193
rs1128249
rs17109256
rs4929927
rs16996700
rs929641
rs4715213
rs9860730
rs1294421
rs10245353
rs2972164
rs1316952
rs17451107
rs4640244
rs10783615
rs1011731
rs11663816
rs2765539
rs1563355
rs2179129
rs12549058
rs3786897
rs1569135
rs1440372
rs2287019
All
−0.015
−0.010
−0.005
0.000
MR leave−one−out sensitivity analysis for
'Waist−to−hip ratio || id:ieu−a−73' on 'Non−cancer illness code self−reported: os
