# Supplementary material for: Causal inference study of plasma proteins and blood metabolites mediating the effect of obesity-related indicators on osteoporosis
Source: Front Endocrinol (Lausanne). 2025 Feb 18;16:1435295. doi: 10.3389/fendo.2025.1435295 (PMC11876022; doi:10.3389/fendo.2025.1435295)
Supplement: Supplementary file 5 [file DataSheet5.zip › mr_leaveoneout_plot_ΦíÇμ╡åΦ¢ïτÖ╜/mr_leaveoneout_plot-18prot-a-3123-ukb-a-87.pdf]

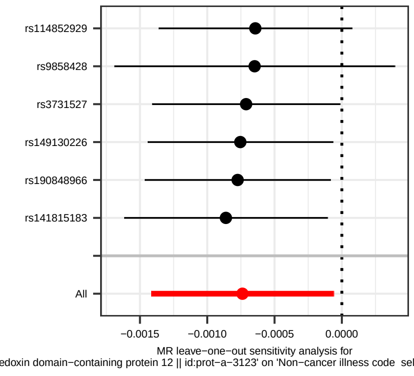
rs114852929
rs9858428
rs3731527
rs149130226
rs190848966
rs141815183
All
−0.0015
−0.0010
−0.0005
0.0000
MR leave−one−out sensitivity analysis for
edoxin domain−containing protein 12 || id:prot−a−3123' on 'Non−cancer illness code sel
