# Supplementary material for: Causal inference study of plasma proteins and blood metabolites mediating the effect of obesity-related indicators on osteoporosis
Source: Front Endocrinol (Lausanne). 2025 Feb 18;16:1435295. doi: 10.3389/fendo.2025.1435295 (PMC11876022; doi:10.3389/fendo.2025.1435295)
Supplement: Supplementary file 5 [file DataSheet5.zip › mr_leaveoneout_plot_ΦíÇμ╡åΦ¢ïτÖ╜/mr_leaveoneout_plot-4prot-a-1458-ukb-a-87.pdf]

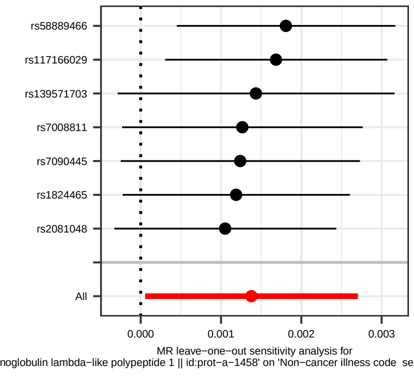

rs58889466
rs117166029
rs139571703
rs7008811
rs7090445
rs1824465
rs2081048
All
0.000
0.001
0.002
0.003
MR leave−one−out sensitivity analysis for
noglobulin lambda−like polypeptide 1 || id:prot−a−1458' on 'Non−cancer illness code se
