# Supplementary material for: Causal inference study of plasma proteins and blood metabolites mediating the effect of obesity-related indicators on osteoporosis
Source: Front Endocrinol (Lausanne). 2025 Feb 18;16:1435295. doi: 10.3389/fendo.2025.1435295 (PMC11876022; doi:10.3389/fendo.2025.1435295)
Supplement: Supplementary file 5 [file DataSheet5.zip › mr_leaveoneout_plot_ΦíÇμ╡åΦ¢ïτÖ╜/mr_leaveoneout_plot-21prot-a-346-ukb-a-87.pdf]

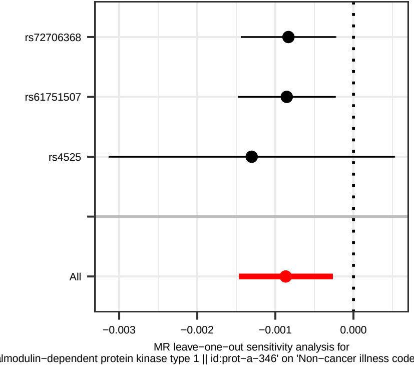

rs72706368
rs61751507
rs4525
All
−0.003
−0.002
−0.001
0.000
MR leave−one−out sensitivity analysis for
almodulin−dependent protein kinase type 1 || id:prot−a−346' on 'Non−cancer illness code
