# Supplementary material for: Causal inference study of plasma proteins and blood metabolites mediating the effect of obesity-related indicators on osteoporosis
Source: Front Endocrinol (Lausanne). 2025 Feb 18;16:1435295. doi: 10.3389/fendo.2025.1435295 (PMC11876022; doi:10.3389/fendo.2025.1435295)
Supplement: Supplementary file 1 [file DataSheet1.zip › Supplementary Figures/Figure notes of Supplementary Figures.doc]

Figure S1. Funnel plot of heterogeneity test for MR of obesity-related indicators (part 1) on osteoporosis.

Figure S2. Funnel plot of heterogeneity test for MR of obesity-related indicators (part 2) on osteoporosis.

Figure S3. Funnel plot of heterogeneity test for MR of plasma proteins in osteoporosis.

Figure S4. Funnel plot of heterogeneity test for MR of uridine in osteoporosis.
